# Suppressed Lone Pair Electrons Explain Unconventional Rise of Lattice Thermal Conductivity in Defective Crystalline Solids

**DOI:** 10.1002/advs.202308075

**Published:** 2024-04-16

**Authors:** Hanhwi Jang, Michael Y. Toriyama, Stanley Abbey, Brakowaa Frimpong, G. Jeffrey Snyder, Yeon Sik Jung, Min‐Wook Oh

**Affiliations:** ^1^ Department of Materials Science and Engineering Korea Advanced Institute of Science and Technology (KAIST) Daejeon 34141 Republic of Korea; ^2^ Department of Materials Science and Engineering Northwestern University Evanston IL 60208 USA; ^3^ Department of Materials Science and Engineering Hanbat National University Yuseong‐gu Daejeon 34158 Republic of Korea

**Keywords:** defect engineering, density functional theory, lone pair electrons, thermal conductivity, thermoelectrics

## Abstract

Manipulating thermal properties of materials can be interpreted as the control of how vibrations of atoms (known as phonons) scatter in a crystal lattice. Compared to a perfect crystal, crystalline solids with defects are expected to have shorter phonon mean free paths caused by point defect scattering, leading to lower lattice thermal conductivities than those without defects. While this is true in many cases, alloying can increase the phonon mean free path in the Cd‐doped AgSnSbSe_3_ system to increase the lattice thermal conductivity from 0.65 to 1.05 W m^−1^ K^−1^ by replacing 18% of the Sb sites with Cd. It is found that the presence of lone pair electrons leads to the off‐centering of cations from the centrosymmetric position of a cubic lattice. X‐ray pair distribution function analysis reveals that this structural distortion is relieved when the electronic configuration of the dopant element cannot produce lone pair electrons. Furthermore, a decrease in the Grüneisen parameter with doping is experimentally confirmed, establishing a relationship between the stereochemical activity of lone pair electrons and the lattice anharmonicity. The observed “harmonic” behavior with doping suggests that lone pair electrons must be preserved to effectively suppress phonon transport in these systems.

## Graphical abstract

The primary role of lone pair electrons (LPEs) in a material is to significantly decrease the lattice thermal conductivity. The introduction of Cd into AgSbSnSe_3_ by replacing Sb sites demonstrates a systematic increase in lattice thermal conductivity due to LPEs consumption. This underscores the crucial aspect of retaining lone pair electrons when designing materials with extremely low lattice thermal conductivity.

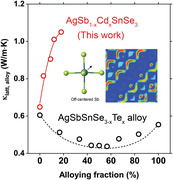

## 1 Introduction

Thermal energy in a semiconducting solid is mostly carried by atomic vibrations, called phonons.^[^ ^1^ ^]^ The thermal conductivity of a material has a finite value because multiple scattering processes result in a finite phonon lifetime.^[^ ^2^ ^]^ In a typical crystalline material, phonon–phonon scattering via Umklapp and normal processes is the dominant mechanism for determining the phonon lifetime.^[^ ^3^ ^]^ With increasing the temperature, the thermal conductivity decreases as the phonon scattering becomes more frequent; therefore, the temperature dependence of the lattice thermal conductivity follows *κ*_latt_ ∼ *T* ^−1^, where *κ*_latt_ and *T* are the lattice thermal conductivity and the absolute temperature, respectively.^[^ ^4^ ^]^ The phonon lifetime is further reduced by the incorporation of impurities into a lattice through point defect scattering.^[^ ^5^ ^]^ The mass fluctuations, lattice strain resulting from the difference in ionic radius between host and guest atoms, or the bonding difference^[^ ^6^ ^]^ have been shown to be effective in reducing *κ*_latt_.^[^ ^7^ ^]^

However, it has been reported that some I–V–VI_2_‐type rock salt compounds show a negligible decrease of *κ*_latt_ with temperature or impurity concentration.^[^ ^8^ ^]^ These observations were in direct contradiction to conventional phonon scattering theory, where increasing temperature or incorporating dopants naturally decreases the phonon mean free path. To explain these anomalies, Nielsen et al. studied the relationship between lone pair electrons (LPEs) and phonon scattering.^[^ ^8a^ ^]^ Briefly, the non‐bonding electrons in the ns^2^ orbital of a group V element can significantly contribute to the high anharmonicity of the lattice. Under this circumstance, the phonon mean free path could be reduced as small as an interatomic distance at room temperature because the high lattice anharmonicity leads to more frequent phonon–phonon scattering.^[^ ^9^ ^]^ Accordingly, the thermal transport becomes diffuson‐mediated rather than a wave‐like phonon, and *κ*_latt_ does not decrease with additional scattering mechanisms.^[^ ^8^, ^10^ ^]^

A natural question arising at this point is what would happen if the amount of LPE changes with the addition of impurities. This is a particularly important question for thermoelectrics because most I‐V‐VI_2_ semiconductors containing LPEs are considered promising thermoelectric materials due to their ultralow *κ*_latt_.^[^ ^11^ ^]^ Often, thermoelectric materials are doped and alloyed with other elements to improve their electrical and thermal properties; however, it would be detrimental to the thermoelectric properties if doping or alloying affects the intrinsic properties of LPEs and significantly increases the *κ*_latt_ values.^[^ ^12^ ^]^ Unfortunately, a study of the relationship between alloying and LPEs has not been well conducted, which hinders a rational understanding and design of LPE‐containing materials with impurities. Therefore, it is imperative to elucidate what happens when external elements perturb the lone pair activity and whether *κ*_latt_ is affected or not.

In this study, we chose AgSbSnSe_3_ as a model system where LPEs dominantly contribute to the phonon scattering, and quantitatively investigated the effect of losing LPEs by doping Cd atoms to the Sb sites. We show that *κ*_latt_ increases from 0.65 to 1.05 W m^−1^ K^−1^ with only 18% substitution of Cd to Sb sites. Using density functional theory (DFT) calculations, we found that LPEs become stereochemically active when cations are locally off‐centered from their centrosymmetric positions. However, the depletion of LPEs by Cd doping relieves the structural distortion to weaken the contribution of LPEs to the lattice anharmonicity, as evidenced by the experimentally measured Grüneisen parameter and the X‐ray pair distribution function (PDF) results. Our results substantiate the importance of preserving LPEs for maintaining ultralow *κ*_latt_ in I‐V‐VI_2_ semiconductors, providing design rules for the development of high‐performance thermoelectrics, thermal barrier coatings, and phase change memories.

## 2 Results and Discussion

### 2.1 Depletion of Lone Pair Electrons by Doping

**Figure 1:**
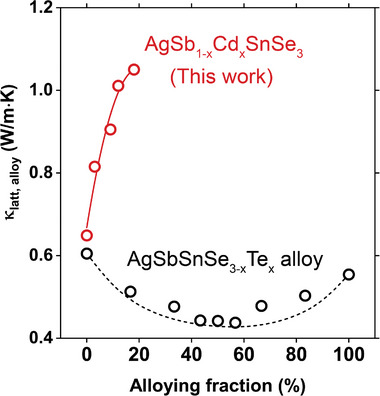
Doping can increase the lattice thermal conductivity. Temperature‐dependent lattice thermal conductivity of AgSbSnSe_3‐x_Te_x_^[^ ^13^ ^]^ (black circles) and AgSb_1‐x_Cd_x_SnSe_3_ (red circles).While Te alloying on Se sites reduces the lattice thermal conductivity, Cd doping on Sb sites persistently increases the lattice thermal conductivity. The dashed black line is the theoretical lattice thermal conductivity expected from the Klemens model. The red line is a guide for the eye.

According to the conventional alloy scattering theory of Klemens, the lattice thermal conductivity of an alloy should follow:

(1) $$\frac{\kappa_{latt,\;alloy}}{\kappa_{0}}=\frac{tan^{-1}u}{u}$$

where *u* is the alloy scattering parameter related to the mass fluctuation of the system.^[^ ^14^ ^]^ Indeed, the experimentally measured lattice thermal conductivity of AgSbSnSe_3‐x_Te_x_ alloys, reported by Luo et al.^[^ ^13^ ^]^ agrees quite well with the values expected by the model (**Figure** **1**, black dots). However, when Cd atoms are incorporated to substitute Sb sites, we observe a sharp increase in κ_*latt*, *alloy*_ with increasing Cd fraction (Figure 1, red dots). We attribute this unconventional increase of *κ*_latt_ to the loss of LPEs with Cd doping, which will be discussed in the following section.

We performed DFT calculations to theoretically understand the effect of Cd doping on Sb sites. Here, we use the *L*1_1_ structure of AgSbSe_2_ to reflect the cation ordering behavior observed in I–V–VI_2_ semiconductors.^[^ ^10^, ^11^, ^15^ ^]^ We show using DFT that LPEs consumed by Cd doping on Sb sites in pristine AgSbSe_2_ causes the increase in *κ*_latt_, and we believe that similar conclusions hold in AgSbSe_2_ alloyed with SnSe.

**Figure 2:**
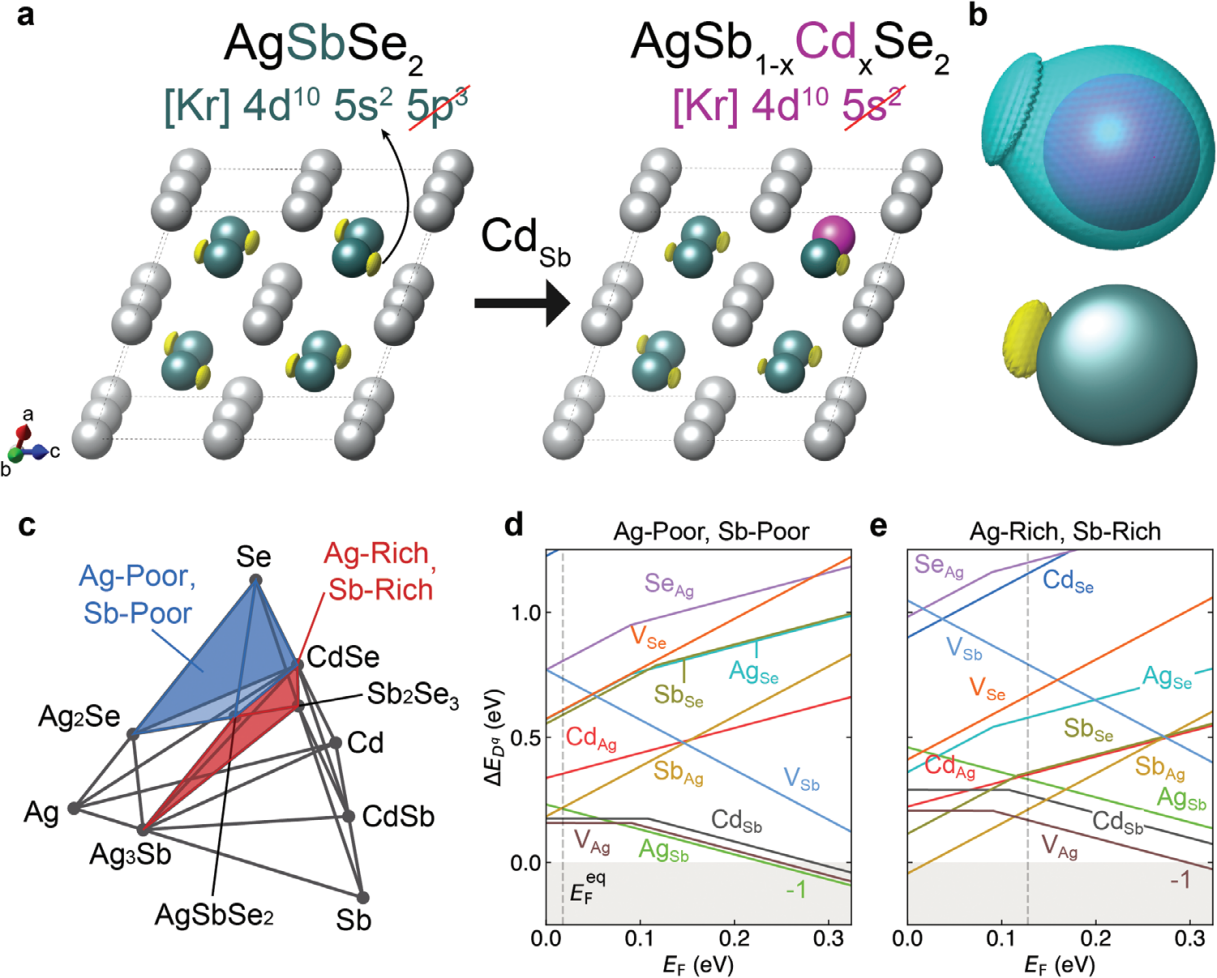
Depletion of lone pair electrons by doping. a) Crystal structure of AgSbSe_2_ and AgSb_1‐x_Cd_x_Se_2_ with the *L*1_1_ ordering. Grey, cyan, and purple atoms denote Ag, Sb, and Cd, respectively. Se atoms are not shown for clarity. The ELF isosurface of ≈0.9 is overlaid to visualize the asymmetric distribution of LPEs near Sb atoms. b) Enlarged view of the ELF isosurfaces. The top panel shows the ELF difference between that of Cd and Sb, while the bottom panel shows the ELF isosurface of Sb. The ELF difference isosurface of the Cd atom is drawn for value of ≈−0.6. c) Calculated quaternary phase diagram of Ag‐Sb‐Cd‐Se system. Blue and red regions denote two representative thermodynamic conditions used to calculate the defect formation energies in (d) and (e). d–e) Calculated point defect formation energies of Cd‐doped AgSbSe_2_ for (d) Ag‐poor / Sb‐poor (when AgSbSe_2_ is in equilibrium with Se, Ag_2_Se, CdSe) and (e) Ag‐rich / Sb‐rich (when in equilibrium with Ag_3_Sb, Sb_2_Se_3_, CdSe) conditions. The formation of Cd_Sb_ is most favored among all possible Cd‐related defects.

**Figure** **2**a shows the electron localization function (ELF) for AgSbSe_2_ and Cd‐doped AgSbSe_2_, which describes the relative degree to which electrons can be found in a spatial region.^[^ ^16^ ^]^ ELF ranges from 0 to 1, where ELF = 1 means electrons are perfectly localized.^[^ ^17^ ^]^ A positive ELF of 0.9 is found near Sb atoms, indicating a strong localization of electrons from two LPEs occupying the 5s orbital. However, the incorporation of Cd into Sb sites significantly reduces the ELF value to 0.25, because all electrons in the 5s orbital of Cd are consumed to form chemical bonds with Se atoms (Figure 2b). The decrease of ELF implies an electron delocalization in Cd–Se bonding compared to Sb–Se.^[^ ^18^ ^]^ Therefore, we can say that Cd doping delocalizes electrons by consuming LPEs in the lattice.

The consumption of LPEs of Sb atoms would most occur when the formation of Cd_Sb_ point defects is most favorable. Therefore, we calculated the formation energies of possible point defects in Cd‐doped AgSbSe_2_ for Ag‐poor/Sb‐poor and Ag‐rich/Sb‐rich conditions (Figure 2d,e), as well as other equilibrium phase regions within the Ag‐Sb‐Se‐Cd quaternary space (Figure 2c; Figure S1, Supporting Information). Our results suggest that Cd occupies the Sb site under any thermodynamic condition, although there are other intrinsic defects (e.g., V_Ag_ and Ag_Sb_) that have lower formation energy than that of Cd_Sb_.

Although there may be a possible discrepancy between the Cd doping in AgSbSe_2_ and AgSbSnSe_3_, our finding that a Cd dopant will most likely substitute on the Sb site in AgSbSe_2_ and deplete LPEs is still relevant to the discussion of defect chemistry in the alloyed system, AgSbSnSe_3_. We can rationalize the defect chemistry in Cd‐doped AgSbSnSe3 by considering both our results and Cd‐doped SnSe. This is because most cation sites in AgSbSnSe_3_ (including Sn) are cubically‐coordinated to Se, similar to the bonding in SnSe where the Sn and Se are bonding in a “distorted” cubic structure. It has been shown that doping polycrystalline SnSe with Cd leads to an increase in the Sn vacancy concentration.^[^ ^19^ ^]^ However, Sn vacancy formation cannot explain our observed increase in lattice thermal conductivity with Cd content due to mass and strain fluctuations induced by vacancies, as well as the formation of other intrinsic point defects (e.g., V_Ag_ and Ag_Sb_).^[^ ^14^ ^]^ Accordingly, the mechanism by which the lattice thermal conductivity increases with Cd content can still be qualitatively explained by Cd substitution on the Sb site, whereby lone pair electrons are depleted from the system.

### 2.2 Lone Pair‐Induced Lattice Distortion

**Figure 3:**
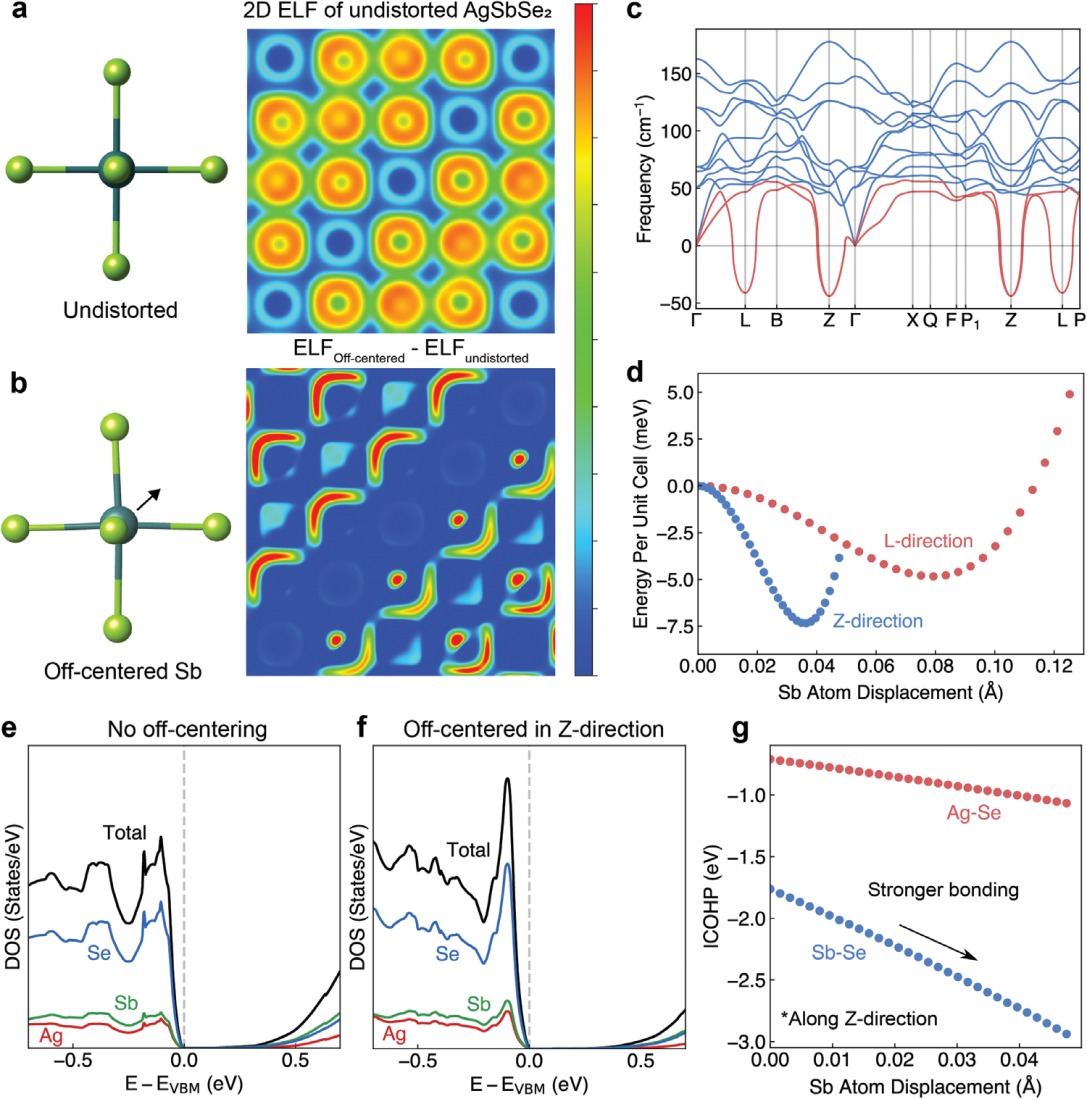
Formation of stereochemically active LPEs with off‐centered cations. Schematic illustration of SbSe_6_ octahedra with corresponding 2D ELF intensity map for a) undistorted and b) Sb off‐centered structures. 2D ELF map for undistorted AgSbSe_2_ was plotted from 0 to 1, ELF difference map from 0 to 0.2. c) Calculated phonon dispersion of AgSbSe_2_ showing multiple imaginary phonon modes. d) Potential energy surface mapping along L‐ and Z‐directions for different Sb atom displacements. e–f) Calculated electronic density of states for e) undistorted and f) Sb off‐centered structures. g) Integrated crystal orbital Hamilton population (ICOHP) for different Sb atom displacements.

We study the off‐centering behavior of cations in the unit cell and its effect on the activity of LPEs. **Figure** **3**a shows the 2D ELF map of AgSbSe_2_, where the Sb atom is located at the centrosymmetric position in SbSe_6_ octahedra. Interestingly, we could not observe any signature of LPEs in undistorted AgSbSe_2_, as the electron distribution near Sb sites appears spherical. This means that two non‐bonding electrons remaining in the 5s orbital of Sb atoms do not necessarily show high ELF values. It is known that cubic‐like materials containing LPEs or local dipoles often show a displacement of the cation from its centrosymmetric position.^[^ ^20^ ^]^ Therefore, it is reasonable to hypothesize that the off‐centering of cations should be related to the activation of LPEs.

In fact, the phonon dispersion curve of undistorted AgSbSe_2_ shows imaginary phonon frequencies, which means that the structure is not dynamically stable and atomic displacements along the certain phonon wavevectors would reduce the free energy of the system (Figure 3c).^[^ ^21^ ^]^ This dynamic instability has been observed in SnTe,^[^ ^22^ ^]^ PbSe,^[^ ^23^ ^]^ and CsSnBr_3_,^[^ ^24^ ^]^ which can be attributed to the local off‐centering of the cations along a certain direction. We, therefore, performed potential energy surface (PES) mapping by displacing atoms corresponding to the vibrational modes along the *L*‐ and *Z*‐directions and found that the total energy could be further reduced by displacing Sb atom for ≈0.04 and 0.08 Å along the *Z*‐ and *L*‐direction, respectively (Figure 3d). This local symmetry breaking by the cation off‐centering in AgSbSe_2_ has also been reported by Dutta et al., where doubly degenerate imaginary phonon modes are observed at only the *L*‐point.^[^ ^25^ ^]^ The difference might be due to the fact that the cation ordering used by Dutta et al. is the *D*4 configuration (space group *Fd*‐3*m*), while we consider the *L*1_1_ ordering (space group *R*‐3*m*). As expected, one can observe an asymmetric bean‐shaped distribution of LPEs in the 2D ELF difference map for the cation off‐centered AgSbSe_2_ (Figure 3b).

We note that this seemingly minor cation off‐centering can affect the electronic structure of AgSbSe_2_. Figure 3e,f shows the atom‐projected electronic density‐of‐states (DOS) of AgSbSe_2_. The DOS near the valence band maximum and the corresponding DOS effective mass vary strongly with the cation off‐centering, suggesting a strong dependence on nearest‐neighbor interactions. This is confirmed by integrated crystal orbital Hamilton population (ICOHP) calculations, where more negative values correspond to stronger bonding strength (Figure 3 g).^[^ ^26^ ^]^ We found that the chemical bonding of both Sb–Se and Ag–Se becomes stronger when Sb is off‐centered. The dynamic instability of the phonon dispersion, as well as the energetics of the chemical bonding, can provide insight into the reason for the cation off‐centering and the corresponding behavior of the LPEs.

### 2.3 Ruling out Possible Scattering Processes

Now, we experimentally verify that the phonon mean free path increases with Cd doping, leading to the increase of *κ*_latt_. It is known that *κ*_latt_ is a product of the heat capacity at a constant volume (*C*_v_), the sound velocity (*v*), and the phonon mean free path (*l*) by the equation:

(2) $$\kappa_{latt}=\frac{1}{3}C_{v}vl$$

The change in *C*_v_ should be marginal for Cd doping according to the Neumann‐Kopp rule, where the heat capacity of a solid substance can be estimated using a weighted average of the heat capacities of the constituting species.^[^ ^27^ ^]^ Therefore, either *v* or *l* would be responsible for the increased *κ*_latt_.

**Figure 4:**
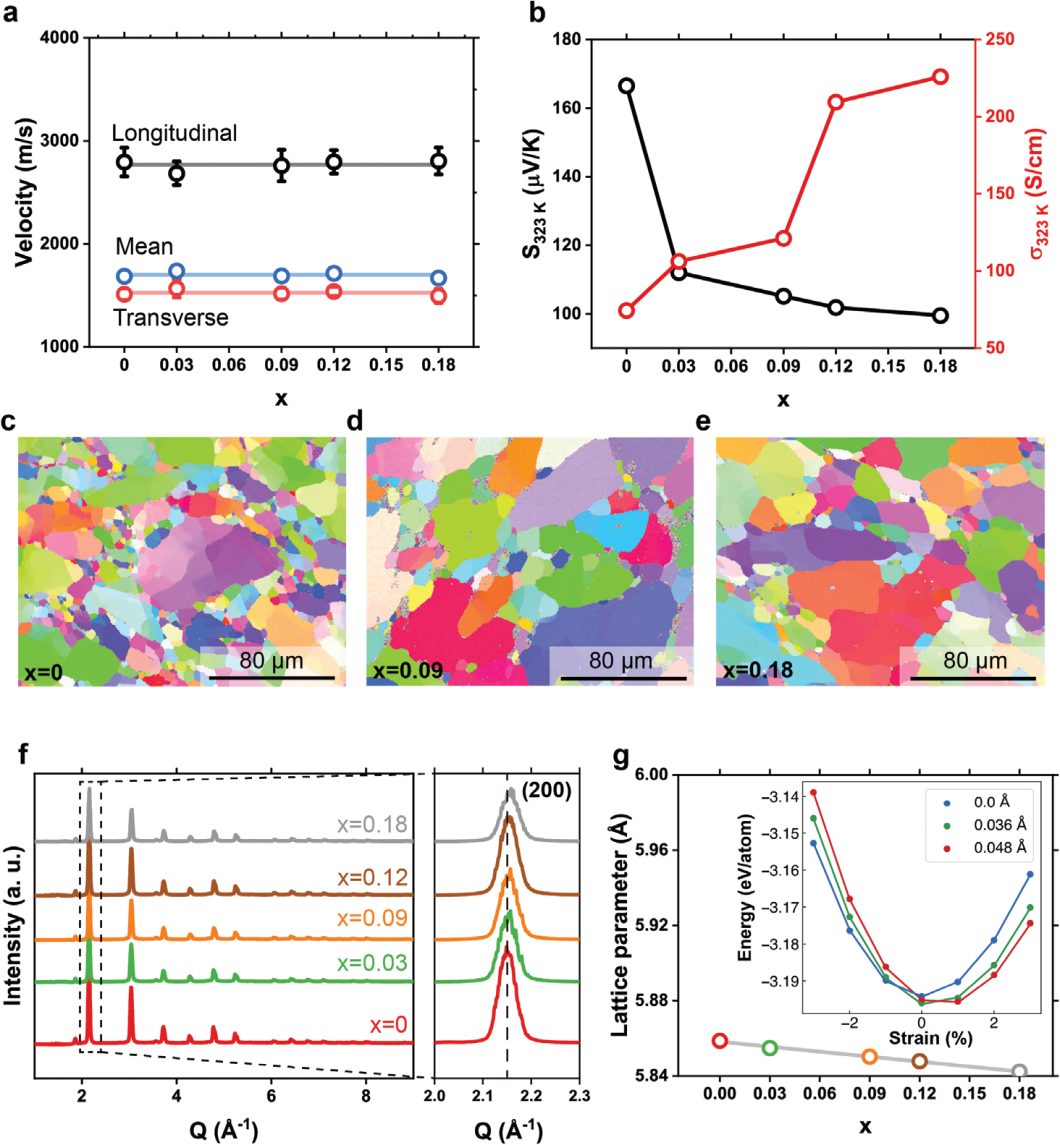
Unaffected phonon group velocity by soluble Cd dopants. a) Measured sound velocity of AgSnSb_1‐x_Cd_x_Se_3_ showing a negligible change. b) Measured Seebeck coefficient and electrical conductivity of AgSnSb_1‐x_Cd_x_Se_3_ c–e) EBSD IPF‐Z images for three samples with different Cd contents. f) Powder X‐ray diffraction pattern of AgSnSb_1‐x_Cd_x_Se_3_. The peak shift of the (200) reflection to higher Q values with increasing x shows a decrease in the lattice parameter with Cd doping. g) Lattice parameters of AgSnSb_1‐x_Cd_x_Se_3_ obtained by Rietveld refinement of (f). Inset is the calculated total energy of the AgSbSe_2_ supercell under tensile and compressive strain for different Sb off‐centering values.

However, we confirmed that the acoustic properties of AgSbSnSe_3_ do not change significantly with Cd doping. **Figure** **4**a shows the measured longitudinal and transverse sound velocities with Cd concentration. The mean velocity was calculated assuming isotropic elastic properties in the polycrystalline samples.^[^ ^11c^ ^]^ It has been reported that the lattice softening due to internal strain^[^ ^4a^ ^]^ or charge carrier^[^ ^28^ ^]^ can significantly reduce the sound velocity and decrease κ_*latt*_. However, the lack of evidence for such phenomena corroborates that the increased phonon mean free path should be responsible for the increased *κ*_latt_.

The phonon mean free path is directly proportional to the relaxation time (τ_*ph*_). Under several phonon scattering mechanisms, the total scattering rate of the phonon ($\tau_{ph}^{-1}$) can be estimated by Matthiessen's rule as follows:

(3) $$\tau_{ph}^{-1}=\tau_{U}^{-1}+\tau_{N}^{-1}+\tau_{PD}^{-1}+\tau_{GB}^{-1}+\tau_{DS}^{-1}+\tau_{DC}^{-1}+\cdots$$

where U, N, PD, GB, DS, and DC denote Umklapp, normal, point defects, grain boundary, dislocation strain, and dislocation core scattering of phonons.^[^ ^5^, ^29^ ^]^ With the addition of impurities, the phonon scattering from point defects, dislocation strain, and dislocation core would decrease the phonon mean free path, thereby not explaining the observed increase in *κ*_latt_. Therefore, it can be suspected that i) promoted grain growth with Cd doping, and/or ii) suppressed Umklapp and normal processes can explain the increased κ_*latt*_.

To exclude the effect of grain boundary scattering on κ_*latt*_, we measured the grain size of AgSnSb_1‐x_Cd_x_Se_3_ (x = 0, 0.09, and 0.18) by electron backscatter diffraction (EBSD) technique. Figure 4c,e show an inverse pole figure along the z‐direction (IPF‐z) color map where the average grain size is estimated to be 7.3, 16, and 18 µm, for x = 0, 0.09, and 0.18 samples, respectively (Figure S2, Supporting Information). It is known that the micron‐sized grains cannot effectively scatter phonons of glass‐like materials, since the phonon mean free path is significantly shorter (a few nanometers) than the grain size.^[^ ^30^ ^]^ Therefore, it is unlikely that the doping‐induced grain growth promoted the increase in *κ*_latt_. Furthermore, by ruling out the grain size effect on κ_*latt*_, only the suppression of Umklapp and normal processes can explain the observed increase in *κ*_latt_.

Powder X‐ray diffraction (XRD) analyses were performed on the synthesized samples to confirm the solubility of Cd in AgSbSnSe_3_ and the absence of secondary phases (Figure 4f). All diffraction peaks were indexed to the rock salt type structure, indicating that secondary phases are not present within the detection limit of XRD.^[^ ^5b^ ^]^ Furthermore, the peak position of the (200) reflection near Q ≈2.15 Å^−1^ shifts persistently to the high‐Q regime with the addition of Cd. The refined lattice parameter from Rietveld analysis shows a linear decrease in Cd concentration, indicating that Cd is soluble in the AgSbSnSe_3_ lattice (Figure 4g). It is interesting to note that the ionic radius of Cd^2+^ (95 pm) is larger than that of Sb^3+^ (76 pm) while the lattice parameter is decreasing.^[^ ^31^ ^]^ We attribute this lattice shrinkage to the consequence of lone pair inactivation. Lattice expansion is generally observed when the dopant with the larger ionic radius substitutes the host atom, but there are some exceptions.^[^ ^32^ ^]^ When LPEs are stereochemically active, the strong electrostatic repulsion of LPEs in cations can repel anions to increase the lattice parameter, explaining an anomalous change of the lattice parameter based on ionic radii.^[^ ^33^ ^]^ For example, the substitution of Sn sites with Sb in BaSn_1‐x_Sb_x_O_3‐δ_ leads to an increase in the lattice parameter,^[^ ^34^ ^]^ while Sn_1‐x_Sb_x_Se shows a persistent decrease with increasing Sb content.^[^ ^35^ ^]^ These reports indicate that the repulsive force extends the bond length to expand the lattice when LPEs are stereochemically active. Reflecting this, DFT calculations (inset in Figure 4g) show that the lattice under tensile strain is preferred compared to the equilibrium lattice constant when the cation off‐centering is 0.048 Å (lone pair active), but the strain is relieved when the off‐centering disappears (lone pair inactive).^[^ ^20c^ ^]^ Given the electrostatically repulsive nature of LPEs, the reason for the preferred tensile strain in the presence of the cation off‐centering is the repulsive force of stereochemically active LPEs, which would be less significant with the depletion of LPEs with Cd doping. Therefore, the decreased lattice parameter with Cd doping is further evidence for lone pair inactivation.

However, the above discussion is not valid when Cd substitutes Ag or Sn sites. Therefore, we measured the electrical transport properties of the samples to analyze the alloying characteristics of Cd impurities (Figure 4b). The Seebeck coefficient of undoped AgSbSnSe_3_ at 323 K is measured to be ≈167 µV K^−1^ and decreases continuously with Cd addition to 100 µV K^−1^ at x = 0.18; conversely, the electrical conductivity increases with x. The positive sign of the Seebeck coefficient indicates that the material is p‐type.^[^ ^36^ ^]^ In addition, the magnitude of the Seebeck coefficient decreases with a carrier concentration.^[^ ^12b^ ^]^ Due to the high volatility of Se, it is possible that the synthesized AgSbSnSe_3_ samples are in the Se‐poor (i.e., Ag‐rich and Sb‐rich) condition, corresponding to the thermodynamic condition of Figure 1e. Under these circumstances, Cd_Sb_ tends to act as an acceptor to increase the hole concentration. Furthermore, elemental quantification using energy‐dispersive X‐ray spectroscopy is consistent with the occupation of Cd on the Sb sites (Figures S3 and S4, Supporting Information). Therefore, our measurements are consistent with the conclusion that Cd substitutes on the Sb site, and thus acts as an acceptor‐like dopant.

### 2.4 Correlating Local Structure, LPEs, and Phonon Transport

**Figure 5:**
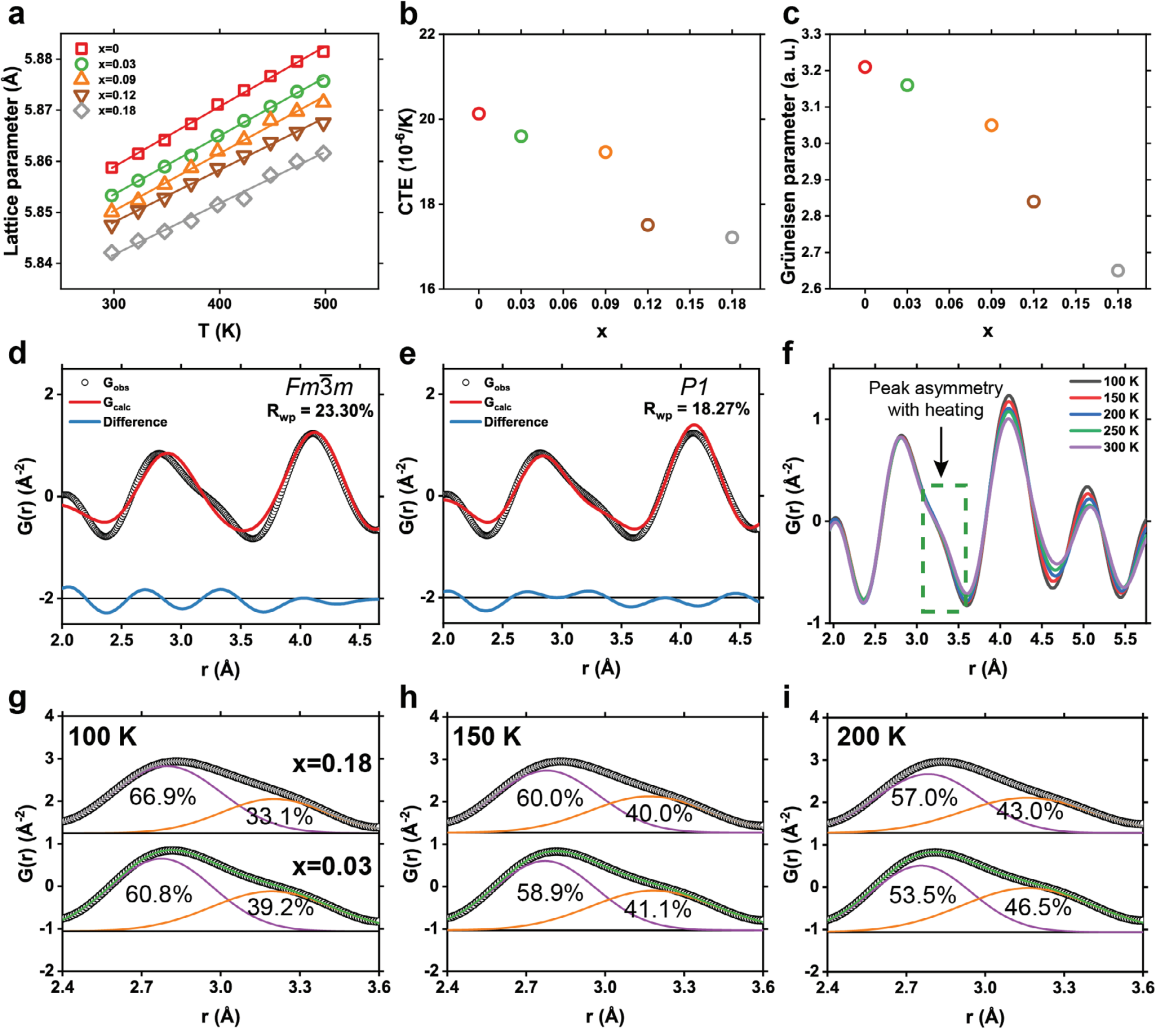
Relieved structural distortion and reduced phonon anharmonicity. a) Temperature‐dependent lattice parameters from in situ powder X‐ray diffraction measurements. b) Coefficient of thermal expansion (CTE) and (c) Grüneisen parameter of AgSnSb_1‐x_Cd_x_Se_3_. d–e) X‐ray pair distribution function (PDF) patterns of the x = 0.03 sample for low *r* values around the first nearest neighbors (≈2.8 Å). The PDF was fitted with the (d) *Fm*‐3*m* and e) *P*1 structure. f) Temperature‐dependent PDF of the x = 0.03 sample measured from 100 to 300 K, showing emphasis because of the presence of stereochemically active LPEs. g–i) Deconvolution of PDFs with two Gaussian fits for x = 0.03 and x = 0.18 samples measured at g) 100 K, h) 150 K, and i) 200 K. Higher Cd concentration suppresses the peak asymmetry due to depleted LPEs.

Now, we verify that the Cd doping does indeed suppress the Umklapp and normal processes of AgSbSnSe_3_ by assessing the degree of anharmonicity of the system. It is widely known that the thermodynamic average Grüneisen parameter can be estimated experimentally as follows:

(4) $$\gamma =\frac{9\alpha BV_{0}}{C}$$

where α, *B*, *V*_0_, and *C* are the linear thermal expansion coefficient, bulk modulus, unit cell volume, and specific heat capacity, respectively.^[^ ^8^, ^37^ ^]^ As shown in **Figure** **5**a, we performed high‐temperature powder XRD analysis to determine α. The lattice parameter increases linearly with temperature and the slope corresponds to the α, which is shown in Figure 5b. The Grüneisen parameter calculated from the experimentally measured values is shown in Figure 5c, showing a continuous drop with increasing Cd concentration. This observation directly supports the fact that the lattice becomes more harmonic with Cd doping, resulting in less frequent phonon scattering and increased phonon mean free path. In other words, Cd doping consumes the LPEs in the lattice, making the cations less off‐centered, and relieving lattice distortion. The change in the phonon mean free path also confirms the role of Cd doping in the depletion of LPEs (Figure S8, Supporting Information). The phonon mean free path for x = 0 was 5.32 ± 1.08 Å, which is close to the interatomic distance of 5.86 Å. According to the Kittel and Clarke models, one should observe the minimum thermal conductivity (the glass limit) as the phonon mean free path approaches the interatomic distance.^[^ ^38^ ^]^ This can be understood by the high Grüneisen parameter of AgSnSbSe_3_ resulting from LPEs that strongly scatter phonons, leading to the glassy thermal conductivity. However, with increasing Cd concentration, we observed a significant increase of the phonon mean free path up to 8.69 ± 1.69 Å at x = 0.18. Since the nanoscale precipitates and point defects typically scatter mid‐ and high‐frequency phonons to reduce the phonon mean free path, the increased phonon mean free path should be interpreted as a result of depleted LPEs, which relieves the cation off‐centering and decrease the Grüneisen parameter.

X‐ray PDF analysis directly shows the change in local structure with the Cd doping. Figure 5d,e shows the PDF of the first nearest neighbor (i.e., cation–Se bonding) of AgSnSb_0.97_Cd_0.03_Se_3_ at 100 K for *r* values smaller than 5 Å, which is particularly important to study the change in local structure.^[^ ^39^ ^]^ We noticed that an unusual peak asymmetry appears near *r* = 3.2 Å, which is related to the cation off‐centering^[^ ^40^ ^]^ or lattice anharmonicity.^[^ ^41^ ^]^ Therefore, attempts to fit the PDF with the fully symmetric *Fm*‐3*m* structure fail to capture this asymmetric feature, while using the *P*1 structure gives better fitting results due to its degrees of freedom to generate an asymmetric peak by local distortion. We also observed the asymmetric peak becomes more pronounced upon heating due to emphasis, a spontaneous symmetry reduction upon heating, which further corroborates the presence of stereochemically active LPEs (Figure 5f).^[^ ^42^ ^]^ This observation naturally leads to the idea that the depletion of LPEs by Cd doping may result in the disappearance of the asymmetric feature of the PDF. Therefore, we compared the relative asymmetry of the PDF in x = 0.03 and x = 0.18 samples by deconvoluting the peak into two Gaussian fits and evaluating their ratio. Figure 5g shows that the orange Gaussian fit (which accounts for the peak asymmetry) becomes less dominant with increasing Cd concentration, which can be attributed to the lower LPE concentration in the x = 0.18 sample. As the temperature is further increased, the peak asymmetry becomes more dominant due to the emphasis; however, the overall tendency remains the same with x = 0.03 showing higher peak asymmetry (Figure 5 h,i). Therefore, the disappearance of the peak tailing suggests that the LPEs become stereochemically inactive and the electrostatic repulsion by them could be alleviated. Indeed, fitting the PDF of x = 0.18 sample with the *Fm*‐3*m* structure gives a lower *R*_wp_ value than that of x = 0.03, indicating Cd doping makes the symmetry of the lattice more cubic‐like by depleting the LPEs. (Figure S14, Supporting Information) We also observed the suppression of low‐energy soft phonon modes with the LPE depletion using Raman spectroscopy, suggesting that reduced LPE concentration is affecting phonon properties (Figure S15, Supporting Information). While the Raman activity of the cubic AgSnSbSe_3_ is not allowed by the group theory,^[^ ^22^, ^25^ ^]^ the appearance of Raman peaks indicates the local cation off‐centering. A further decrease in the Raman intensity suggests that the lattice becomes a more cubic‐like structure with weaker Raman activity. Overall, these results indicate that the unconventional increase of *κ*_latt_ originates from the suppressed Umklapp process, evidenced by the decreased Grüneisen parameter, is attributed to the consumed LPEs from Cd doping to Sb sites.

## 3 Conclusions

We have shown that the depletion of LPEs upon doping can be responsible for the increased *κ*_latt_ in lone pair‐containing materials. DFT calculations revealed that the lone pair activity is observed with cation off‐centering, leading to the lattice distortion and contributing to the high lattice anharmonicity. While anion alloying does not affect the configuration of LPEs, cation doping to the group V element in I–V–VI_2_ semiconductors can consume existing LPEs; accordingly, *κ*_latt_ decreases with anion alloying but increases with cation alloying. We experimentally demonstrated the reduced lattice anharmonicity and relieved structural distortion with cation doping to corroborate that removing LPEs can immediately increase the phonon mean free path. The findings of our work would be significant not only for the fundamental understanding of the thermal behavior of materials but also for providing design rules for the development of I–V–VI_2_‐based functional thermal materials.

## 4 Experimental Section

### Materials

Ag shots (99.99%, Alfa Aesar), Sb shots (99.999%, 5N Plus), Cd shots (99.999%, Alfa Aesar), Sn shots (99.999%, American Elements), and Se shots (99.999%, Alfa Aesar) were used without further purification.

### Synthesis

Conventional melting and solidification were used process to synthesize polycrystalline AgSnSb_1‐x_Cd_x_Se_3_ (x = 0, 0.03, 0.06, 0.09, 0.12, 0.18) samples. Stoichiometric amounts of elements were weighed and placed in a carbon‐coated quartz tube. Then, the tube was flame‐sealed under a high vacuum (≤10^−4^ Torr) and placed in a vertical tube furnace. The elements were melted at 1273 K for 6 h, annealed at 773 K for 24 h, and cooled to room temperature using water quenching.

### Densification

The solidified ingots were pulverized using agate mortar and pestle. An induction hot‐pressing technique for rapid consolidation of the samples was used with a ramping rate higher than 50 K/min.^[^ ^43^ ^]^ The pulverized powder was placed in a graphite mold and pressed using graphite punches having a diameter of 12.7 mm. Then, the sintering was conducted under a uniaxial pressure of 50 MPa at 773 K for 10 min under an argon atmosphere. During the sintering, the temperature of the die was constantly monitored with thermocouples.

### Transport Property Measurement

The high‐temperature electrical conductivity was measured using a home‐built Hall effect measurement system under a high vacuum (≤10^−4^ Torr).^[^ ^44^ ^]^ The sample contact was established using pressure‐assisted molybdenum contacts with the van der Pauw configuration. The Hall coefficient (*R*_H_) was measured simultaneously using an excitation current of 100 mA under a magnetic field of 2T generated from a water‐cooled electromagnet. The carrier concentration was calculated using the equation *n*_H_ = 1/e*R*_H_. The high hole concentration of the sample (≈10^20^ cm^−3^) confirms that the sample was in the degenerate regime, and the contribution of bipolar conduction to the thermal conductivity might be minimal (Figure S13, Supporting Information). The high‐temperature Seebeck coefficient was measured using chrome‐niobium thermocouples by oscillating temperature gradient within 7 K under a high vacuum.^[^ ^45^ ^]^ Thermal conductivity was estimated from the product of the density (*ρ*), specific heat capacity (*C*_P_), and thermal diffusivity (*D*). *D* was measured using the laser flash apparatus (LFA 457, Netzsch Ltd.). Both sides of the samples were coated with a thin graphite layer to improve the sample emissivity. *C*_P_ was estimated using the Dulong‐Petit law. *ρ* was determined using an Archimedes method.

### X‐ray Diffraction Measurement

Temperature‐dependent X‐ray diffraction (XRD) and pair distribution function (PDF) measurements were conducted using a STOE StadiVari diffractometer. It was known that a laboratory X‐ray diffractometer with Ag K_α_ radiation source can provide satisfactory PDF measurement data due to high *Q*_max_ value it can reach. Therefore, the spurious PDF peak broadening and peak shift were less frequent.^[^ ^46^ ^]^ An AXO Ag K_α_ micro‐focus sealed X‐ray A‐MiXS source (λ = 0.560834 Å), running at 65 kV and 0.68 mA, and a Dectris Pilatus3 R CdTe 300K Hybrid Photon Counting detector were used. The powder was sieved under 25 µm and packed in a glass capillary tube with a diameter of 1 mm. The capillary was flame‐sealed under a vacuum. The temperature of the samples was controlled with an Oxford Cryosystems low‐temperature device, and the exposure time for each frame was 3600 s. The collection angle (2*θ*) ranges from 5° to 164° for PDF measurement, and 5° to 40° for high‐temperature XRD measurement. Background signals were subtracted by measuring empty capillaries under the same measurement condition. The calibration was performed using LaB_6_ as a standard reference. PDF data reduction and structural refinement were performed using PDFgetx3 and PDFgui, respectively.^[^ ^47^ ^]^ The off‐centered *L*1_1_ structure obtained from phonon calculation was used to calculate the theoretical PDF pattern of distorted cubic, while *Fm*‐3*m* structure was used to model fully symmetric cubic. The refinement parameters were the scale factor, atomic positions, dynamic correlation factor (delta 2), lattice parameter, and isotropic thermal displacement parameters (*U*_iso_).

### Characterization

The polished surface of the sample was analyzed using a field emission scanning electron microscope (SU‐8230, Hitachi) at an acceleration voltage of 20 kV. Elemental quantification and mapping were performed using energy‐dispersive X‐ray spectroscopy (EDS) equipped with the SEM instrument. Silicon drift detectors (SDDs) with silicon nitride windows (Octane Elite Plus, EDAX) were used to acquire the EDS spectra, and the spectra were processed using APEX software. Backscattered electron (BSE) imaging was performed using a semiconductor‐type photodiode BSE detector equipped in the same apparatus. The signal for EDS elemental mapping was acquired for more than 5 min to achieve a high signal‐to‐noise ratio. Ultrafine sample surfaces were prepared by cooling cross‐section polisher (IB‐19520CCP, JEOL) using Ar^+^ ion accelerated at 5 kV. The milling was conducted at 193 K using a liquid nitrogen‐cooled sample stage to minimize possible structural deformation during ion milling. The electron backscatter diffraction (EBSD) image was acquired using an environmental scanning electron microscope (Quattro S, Thermo Fisher) equipped with EDAX Velocity EBSD camera. Raman spectra were acquired at room temperature using a Raman spectrometer (XperRAM S, Nanobase). A 532 nm laser was used with a power of 0.54 mW and a diameter of 1 µm under a ×40 magnification. Raman spectra were measured using gratings with 1800 grooves/mm.

### Sound Velocity Measurement

The pulse‐echo method was used to measure the speed of sound of AgSnSb_1‐x_Cd_x_Se_3_ samples. Briefly, the piezoelectric transducer initially sends an acoustic stress wave to the sample, and the signal was received by an identical transducer. Longitudinal and transverse transducers with a center frequency of 5 MHz (Olympus V1091 and V157‐RM) and a Tektronic TBS 1072B‐EDU oscilloscope were used to record the waveforms. The time delay, *t*_d_, between back‐wall echoes was determined by varying *t*_d_ to maximize the cross‐correlation of the two reflections.^[^ ^4a^ ^]^ The sound velocity was calculated as *v* = 2*d*/*t*_d_, where *d* is the sample thickness. The average sound velocity was calculated by assuming isotropic elastic properties in the polycrystalline samples as follows: $v_{avg}={\left[\frac{1}{3}\left(\frac{1}{v_{L}^{3}}+\frac{2}{v_{T}^{3}}\right)\right]}^{-1/3}$, where *v*_L_ and *v*_T_ are the measured longitudinal and transverse velocities, respectively. The uncertainty in *v_avg_* was estimated by $\Delta v_{avg}=\sqrt{{\left(\frac{\partial v_{avg}}{\partial v_{L}}\Delta v_{L}\right)}^{2}+{\left(\frac{\partial v_{avg}}{\partial v_{T}}\Delta v_{T}\right)}^{2}}$, where Δ is an uncertainty in the measured variable.

### Density Functional Theory Calculations

First‐principles density functional theory (DFT) calculations were performed using the Vienne Ab initio Simulation Package (VASP).^[^ ^48^ ^]^ The projector augmented wave (PAW) method^[^ ^49^ ^]^ was used to treat interactions between core and valence electrons. The Perdew‐Burke‐Ernzerhof (PBE) exchange‐correlation functional,^[^ ^50^ ^]^ within the generalized gradient approximation (GGA), was employed. A rotationally‐invariant Hubbard *U* correction of 5 eV was applied to Ag.^[^ ^51^ ^]^ Spin‐orbit interactions were included in all total energy calculations.^[^ ^52^ ^]^ Unless otherwise stated, an energy cutoff of 400 eV was used.

The standard supercell approach^[^ ^53^ ^]^ was used to calculate the formation energies of point defects in AgSbSe_2_. A 64‐atom supercell of the *L*1_1_ ordering^[^ ^11d^ ^]^ was considered in all defect formation energy calculations. The calculations were automated by the pylada‐defects software.^[^ ^54^ ^]^ A 2×2×2 Γ‐centered *k*‐point mesh^[^ ^55^ ^]^ was used in all defect calculations.^[^ ^56^ ^]^ The defect formation energy ($\Delta E_{D^{q}}$) of a defect *D* with charge state *q* can be calculated as

(5) $$\Delta E_{D^{q}}=E_{D^{q}}-E_{H}-\sum_{i}n_{i}\mu_{i}+qE_{F}+E_{Corr}$$

where $E_{D^{q}}$ and *E_H_* are the total energies of the supercell with and without the defect, respectively. The chemical potential $\mu_{i}=\mu_{i}^{0}+\Delta \mu_{i}$ of atom *i* has contributions from an intrinsic reference energy $\mu_{i}^{0}$ and from thermodynamic conditions of the system Δε_*i*_. The reference energies $\mu_{i}^{0}$ were fitted to experimental formation enthalpies under standard conditions of compounds within the Ag‐Sb‐Se‐Cd quaternary phase space (Table S1, Supporting Information).^[^ ^51b^ ^]^ The bounds of Δε_*i*_ were determined from the formation enthalpies such that AgSbSe_2_ was stable with respect to competing phases (Table S2, Supporting Information). The chemical potential contribution to the formation energy was determined by whether an atom was added (*n_i_* > 0) or removed (*n_i_* < 0) to create defect *D*. The Fermi energy *E_F_* ranges between the valence band maximum and conduction band minimum, compelling an accurate measure of the band edge energies. Due to the well‐known underestimation of the band gap in GGA‐type functionals, a shift to the band edge positions according to quasiparticle GW calculations was applied,^[^ ^52a^ ^]^ resulting in a band gap of 0.324 eV (Figure S5, Supporting Information). Energy corrections (*E_Corr_*) were calculated according to the methodology of Lany and Zunger.^[^ ^56^ ^]^

Phonon calculations were performed using the phonopy software.^[^ ^57^ ^]^ A stringent energy cutoff of 650 eV was used in all phonon calculations. The potential energy surfaces at the L‐ and Z‐points, where unstable phonon modes exist, were calculated by modulating a 2×2×2 supercell containing 32 atoms.^[^ ^58^ ^]^ The integrated COHP calculations were performed on the modulated structures using version 4.0.0 of the LOBSTER software.^[^ ^59^ ^]^

## Conflict of Interest

The authors declare no conflict of interest.

## Supporting information

Supporting Information

## Data Availability

The data that support the findings of this study are available from the corresponding author upon reasonable request.

## References

[1] a) P. Acharyya , T. Ghosh , K. Pal , K. S. Rana , M. Dutta , D. Swain , M. Etter , A. Soni , U. V. Waghmare , K. Biswas , Nat. Commun. 2022, 13, 5053; 36030224 10.1038/s41467-022-32773-4 PMC9420152

[2] M. Christensen , A. B. Abrahamsen , N. B. Christensen , F. Juranyi , N. H. Andersen , K. Lefmann , J. Andreasson , C. R. H. Bahl , B. B. Iversen , Nat. Mater. 2008, 7, 811. 18758454 10.1038/nmat2273

[3] D. L. Nika , E. P. Pokatilov , A. S. Askerov , A. A. Balandin , Phys. Rev. B 2009, 79, 155413.

[4] a) R. Hanus , M. T. Agne , A. J. E. Rettie , Z. Chen , G. Tan , D. Y. Chung , M. G. Kanatzidis , Y. Pei , P. W. Voorhees , G. J. Snyder , Adv. Mater. 2019, 31, 1900108; 10.1002/adma.201900108 30968467

[5] a) R. Gurunathan , R. Hanus , M. Dylla , A. Katre , G. J. Snyder , Phys. Rev. Appl. 2020, 13, 034011;

[6] a) M. Dutta , K. Pal , U. V. Waghmare , K. Biswas , Chem. Sci. 2019, 10, 4905; 31183040 10.1039/c9sc00485h PMC6521233

[7] a) L. Fu , M. Yin , D. Wu , W. Li , D. Feng , L. Huang , J. He , Energy Environ. Sci. 2017, 10, 2030;

[8] a) M. D. Nielsen , V. Ozolins , J. P. Heremans , Energy Environ. Sci. 2013, 6, 570;

[9] R. Wolfe , J. H. Wernick , S. E. Haszko , J. Appl. Phys. 2004, 31, 1959.

[10] J. Ma , O. Delaire , A. F. May , C. E. Carlton , M. A. Mcguire , L. H. Vanbebber , D. L. Abernathy , G. Ehlers , T. Hong , A. Huq , W. Tian , V. M. Keppens , Y. Shao‐Horn , B. C. Sales , Nat. Nanotechnol. 2013, 8, 445. 23728075 10.1038/nnano.2013.95

[11] a) S. Roychowdhury , T. Ghosh , R. Arora , M. Samanta , L. Xie , N. K. Singh , A. Soni , J. He , U. V. Waghmare , K. Biswas , Science 2021, 371, 722; 33574210 10.1126/science.abb3517

[12] a) T. Zhu , Y. Liu , C. Fu , J. P. Heremans , J. G. Snyder , X. Zhao , Adv. Mater. 2017, 29, 1605884; 10.1002/adma.201605884 28262991

[13] Y. Luo , S. Hao , S. Cai , T. J. Slade , Z. Z. Luo , V. P. Dravid , C. Wolverton , Q. Yan , M. G. Kanatzidis , J. Am. Chem. Soc. 2020, 142, 15187. 32786784 10.1021/jacs.0c07803

[14] R. Gurunathan , R. Hanus , G. J. Snyder , Mater. Horiz. 2020, 7, 1452.

[15] a) E. Quarez , K.‐F. Hsu , R. Pcionek , N. Frangis , E. K. Polychroniadis , M. G. Kanatzidis , J. Am. Chem. Soc. 2005, 127, 9177; 15969596 10.1021/ja051653o

[16] B. Ge , C. Li , W. Lu , H. Ye , R. Li , W. He , Z. Wei , Z. Shi , D. Kim , C. Zhou , M. Zhu , M. Wuttig , Y. Yu , Adv. Energy Mater. 2023, 13, 2300965.

[17] Z. Liu , W. Zhang , W. Gao , T. Mori , Energy Environ. Sci. 2021, 14, 3579.

[18] S. Berski , G. Gajewski , Z. Latajka , J. Mol. Struct. 2007, 844–845, 278.

[19] X. Shi , A. Wu , T. Feng , K. Zheng , W. Liu , Q. Sun , M. Hong , S. T. Pantelides , Z.‐G. Chen , J. Zou , Adv. Energy Mater. 2019, 9, 1803242.

[20] a) Y. Zhang , X. Ke , P. R. C. Kent , J. Yang , C. Chen , Phys. Rev. Lett. 2011, 107, 175503; 22107535 10.1103/PhysRevLett.107.175503

[21] S. Zhang , J. Zhou , Q. Wang , X. Chen , Y. Kawazoe , P. Jena , Proc. Natl. Acad. Sci. USA 2015, 112, 2372. 25646451 10.1073/pnas.1416591112 PMC4345574

[22] A. Banik , T. Ghosh , R. Arora , M. Dutta , J. Pandey , S. Acharya , A. Soni , U. V. Waghmare , K. Biswas , Energy Environ. Sci. 2019, 12, 589.

[23] S. Cai , S. Hao , Z.‐Z. Luo , X. Li , I. Hadar , T. P. Bailey , X. Hu , C. Uher , Y.‐Y. Hu , C. Wolverton , V. P. Dravid , M. G. Kanatzidis , Energy Environ. Sci. 2020, 13, 200.

[24] H. Xie , S. Hao , J. Bao , T. J. Slade , G. J. Snyder , C. Wolverton , M. G. Kanatzidis , J. Am. Chem. Soc. 2020, 142, 9553. 32320237 10.1021/jacs.0c03427

[25] M. Dutta , M. V. D. Prasad , J. Pandey , A. Soni , U. V. Waghmare , K. Biswas , Angew. Chem., Int. Ed. 2022, 61, e202200071. 10.1002/anie.202200071 35137508

[26] a) R. Dronskowski , P. E. Bloechl , J. Phys. Chem. 1993, 97, 8617;

[27] J. Leitner , P. Vonka , D. Sedmidubský , P. Svoboda , Thermochim. Acta 2010, 497, 7.

[28] T. J. Slade , S. Anand , M. Wood , J. P. Male , K. Imasato , D. Cheikh , M. M. Al Malki , M. T. Agne , K. J. Griffith , S. K. Bux , C. Wolverton , M. G. Kanatzidis , G. J. Snyder , Joule 2021, 5, 1168.

[29] S. I. Kim , K. H. Lee , H. A. Mun , H. S. Kim , S. W. Hwang , J. W. Roh , D. J. Yang , W. H. Shin , X. S. Li , Y. H. Lee , G. J. Snyder , S. W. Kim , Science 2015, 348, 109. 25838382 10.1126/science.aaa4166

[30] a) X. J. Zheng , L. Zhu , Y.‐H. Zhou , Q. Zhang , Appl. Phys. Lett. 2005, 87, 242101;

[31] R. D. Shannon , Acta Crystallogr. A 1976, 32, 751.

[32] a) G. H. Olsen , M. H. Sørby , S. M. Selbach , T. Grande , Chem. Mater. 2017, 29, 6414;

[33] a) F. Goutenoire , O. Isnard , E. Suard , O. Bohnke , Y. Laligant , R. Retoux , P. Lacorre , J. Mater. Chem. 2001, 11, 119;

[34] a) R. J. Cava , P. Gammel , B. Batlogg , J. J. Krajewski , W. F. Peck , L. W. Rupp , R. Felder , R. B. Van Dover , Phys. Rev. B 1990, 42, 4815; 10.1103/physrevb.42.4815 9996029

[35] K. Monikapani , V. Vijay , R. Abinaya , J. Archana , S. Harish , M. Navaneethan , J. Alloys Compd. 2022, 923, 165961.

[36] J.‐J. Gu , M.‐W. Oh , H. Inui , D. Zhang , Phys. Rev. B 2005, 71, 113201.

[37] A. M. Hofmeister , H.‐K. Mao , Proc. Natl. Acad. Sci. USA 2002, 99, 559.11805314

[38] M. T. Agne , R. Hanus , G. J. Snyder , Energy Environ. Sci. 2018, 11, 609.

[39] M. Dutta , K. Pal , M. Etter , U. V. Waghmare , K. Biswas , J. Am. Chem. Soc. 2021, 143, 16839. 34606248 10.1021/jacs.1c08931

[40] J. Dong , Y. Jiang , Y. Sun , J. Liu , J. Pei , W. Li , X. Y. Tan , L. Hu , N. Jia , B. Xu , Q. Li , J.‐F. Li , Q. Yan , M. G. Kanatzidis , J. Am. Chem. Soc. 1988, 145, 2023. 10.1021/jacs.2c12877 36648753

[41] C. W. Li , J. Ma , H. B. Cao , A. F. May , D. L. Abernathy , G. Ehlers , C. Hoffmann , X. Wang , T. Hong , A. Huq , O. Gourdon , O. Delaire , Phys. Rev. B 2014, 90, 214303.

[42] D. H. Fabini , G. Laurita , J. S. Bechtel , C. C. Stoumpos , H. A. Evans , A. G. Kontos , Y. S. Raptis , P. Falaras , A. Van Der Ven , M. G. Kanatzidis , R. Seshadri , J. Am. Chem. Soc. 2016, 138, 11820. 27583813 10.1021/jacs.6b06287

[43] A. D. LaLonde , T. Ikeda , G. J. Snyder , Rev. Sci. Instrum. 2011, 82, 025104. 21361630 10.1063/1.3534080

[44] K. A. Borup , E. S. Toberer , L. D. Zoltan , G. Nakatsukasa , M. Errico , J.‐P. Fleurial , B. B. Iversen , G. J. Snyder , Rev. Sci. Instrum. 2012, 83, 123902. 23278000 10.1063/1.4770124

[45] S. Iwanaga , E. S. Toberer , A. LaLonde , G. J. Snyder , Rev. Sci. Instrum. 2011, 82, 063905. 21721707 10.1063/1.3601358

[46] S. L. J. Thomae , N. Prinz , T. Hartmann , M. Teck , S. Correll , M. Zobel , Rev. Sci. Instrum. 2019, 90, 043905. 31043011 10.1063/1.5093714

[47] a) P. Juhás , T. Davis , C. L. Farrow , S. J. L. Billinge , J. Appl. Crystallogr. 2013, 46, 560;

[48] a) G. Kresse , J. Hafner , Phys. Rev. B 1993, 47, 558; 10.1103/physrevb.47.558 10004490

[49] a) P. E. Blöchl , Phys. Rev. B 1994, 50, 17953; 10.1103/physrevb.50.17953 9976227

[50] J. P. Perdew , K. Burke , M. Ernzerhof , Phys. Rev. Lett. 1996, 77, 3865. 10062328 10.1103/PhysRevLett.77.3865

[51] a) S. L. Dudarev , G. A. Botton , S. Y. Savrasov , C. J. Humphreys , A. P. Sutton , Phys. Rev. B 1998, 57, 1505;

[52] a) A. Goyal , P. Gorai , E. S. Toberer , V. Stevanovic , npj Comput. Mater. 2017, 3, 42;

[53] a) C. Freysoldt , B. Grabowski , T. Hickel , J. Neugebauer , G. Kresse , A. Janotti , C. G. Van De Walle , Rev. Mod. Phys. 2014, 86, 253;

[54] A. Goyal , P. Gorai , H. Peng , S. Lany , V. Stevanovic , Comput. Mater. Sci. 2017, 130, 1.

[55] H. J. Monkhorst , J. D. Pack , Phys. Rev. B 1976, 13, 5188.

[56] S. Lany , A. Zunger , Phys. Rev. B 2008, 78, 235104.

[57] a) A. Togo , I. Tanaka , Scr. Mater. 2015, 108, 1;

[58] A. Togo , I. Tanaka , Phys. Rev. B 2013, 87, 184104.

[59] a) S. Maintz , V. L. Deringer , A. L. Tchougréeff , R. Dronskowski , J. Comput. Chem. 2016, 37, 1030; 26914535 10.1002/jcc.24300 PMC5067632

