# Supplementary material for: Suppressed Lone Pair Electrons Explain Unconventional Rise of Lattice Thermal Conductivity in Defective Crystalline Solids
Source: Adv Sci (Weinh). 2024 Apr 16;11(24):2308075. doi: 10.1002/advs.202308075 (PMC11200014; doi:10.1002/advs.202308075)
Supplement: Supplementary file 1 — Supporting Information [file ADVS-11-2308075-s001.pdf]

ADVANCED SCIENCE
Open Access

Supporting Information

for *Adv. Sci.*, DOI 10.1002/advs.202308075

Suppressed Lone Pair Electrons Explain Unconventional Rise of Lattice Thermal Conductivity in Defective Crystalline Solids

Hanhwi Jang, Michael Y. Toriyama, Stanley Abbey, Brakowaa Frimpong, G. Jeffrey Snyder, Yeon Sik Jung* and Min-Wook Oh**

Supporting Information

Suppressed Lone Pair Electrons Explain Unconventional Rise of Lattice Thermal Conductivity in Defective Crystalline Solids

Hanhwi Jang, Michael Y. Toriyama, Stanley Abbey, Brakowaa Frimpong, G. Jeffrey Snyder, Yeon Sik Jung*, and Min-Wook Oh**

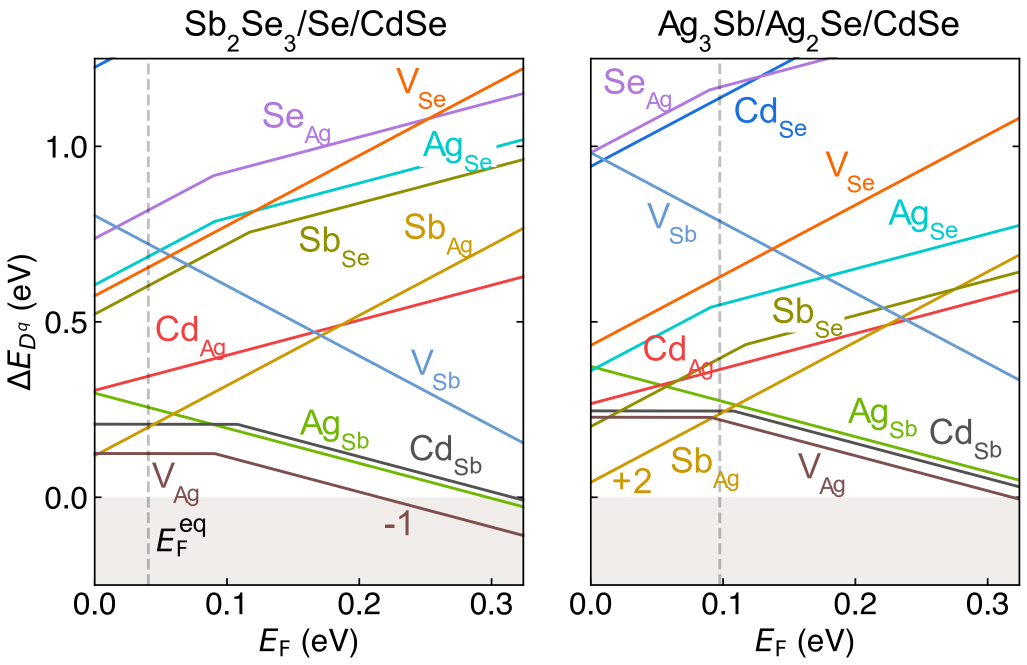

Supplementary Figure 1| Calculated point defect formation energies with various equilibrium phases.

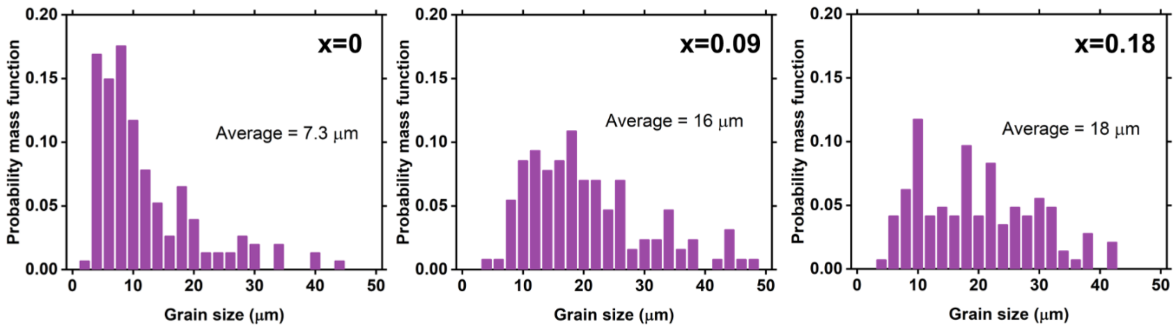

Supplementary Figure 2| The distribution of grain size for different Cd concentrations.

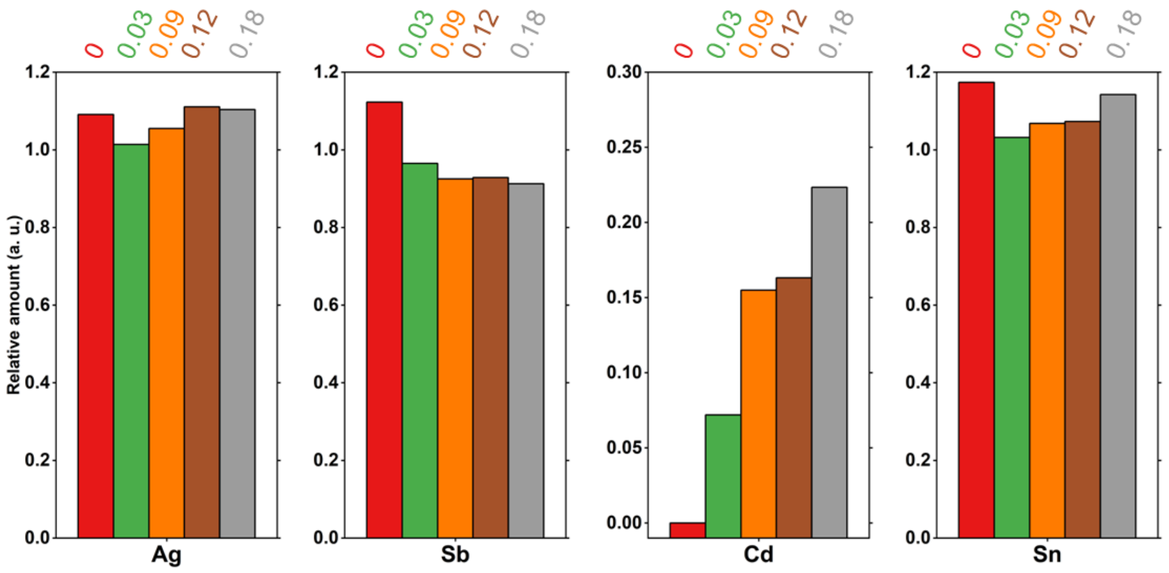

Supplementary Figure 3| SEM-EDS elemental quantification. Quantitative analysis for $AgSb_{1-x}Cd_xSnSe_3$. The numbers in the upper panel indicate the Cd concentration in the sample. All concentrations were normalized to the Se concentration.

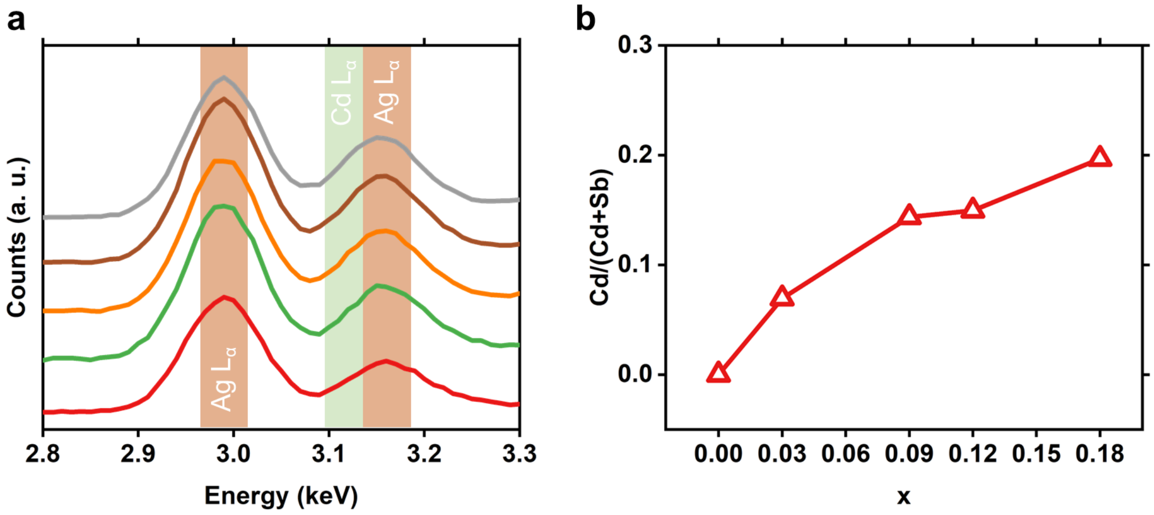

Supplementary Figure 4| SEM-EDS spectra and relative ratio of Cd. (a) EDS spectra for $AgSb_{1-x}Cd_xSnSe_3$ samples for 2.8 to 3.3 keV. The Cd L_α signal corresponds to the 3.13 keV. (b) Calculated Cd concentration normalized to the occupancy of Sb sites.

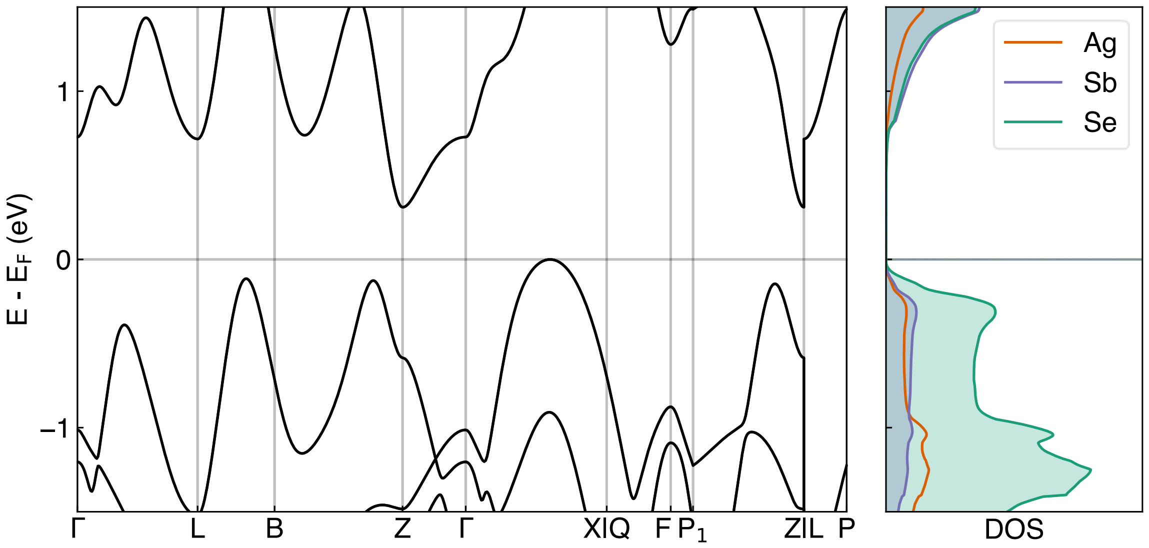

Supplementary Figure 5| Calculated electronic band structure and density-of-states of $AgSbSe_2$ with $L1_1$ ordering. Quasiparticle GW calculations were applied to correct the band gap underestimation of GGA-type calculations.

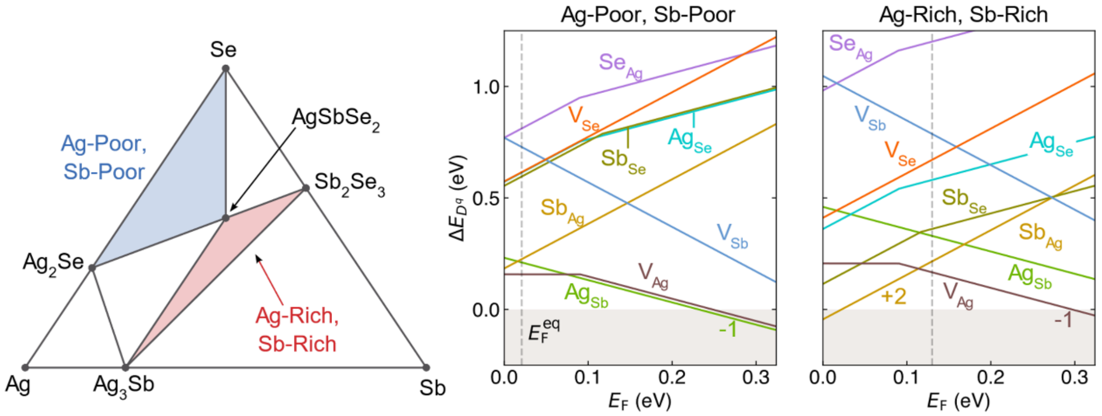

Supplementary Figure 6| Calculated ternary phase diagram of Ag-Sb-Se system and corresponding point defect formation energies.

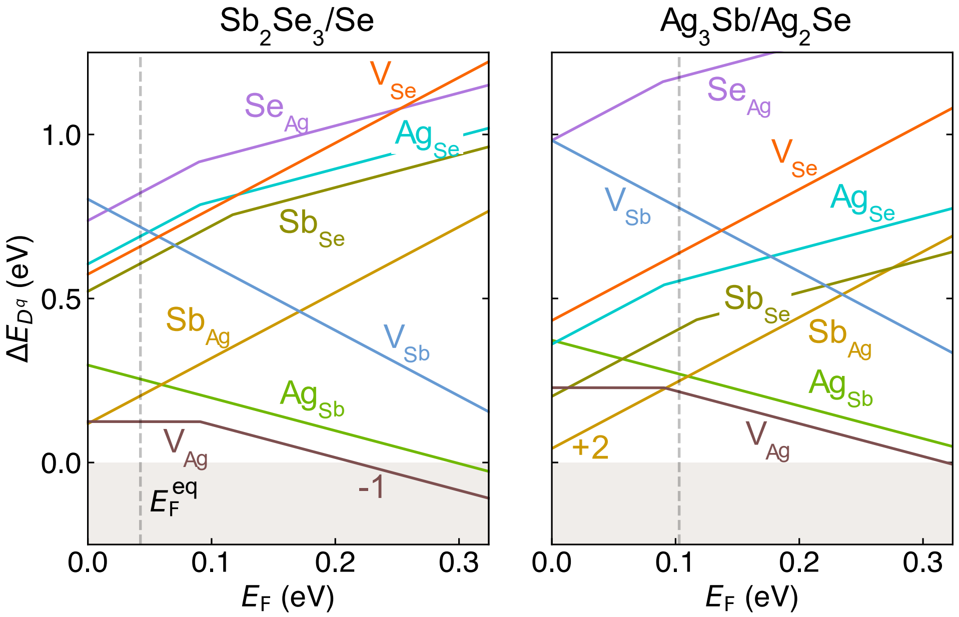

Supplementary Figure 7| Calculated point defect formation energies when the equilibrium phase of $AgSbSe_2$ are (left) Sb_2Se/Se and (right) Ag_3Sb/Ag_2Se.

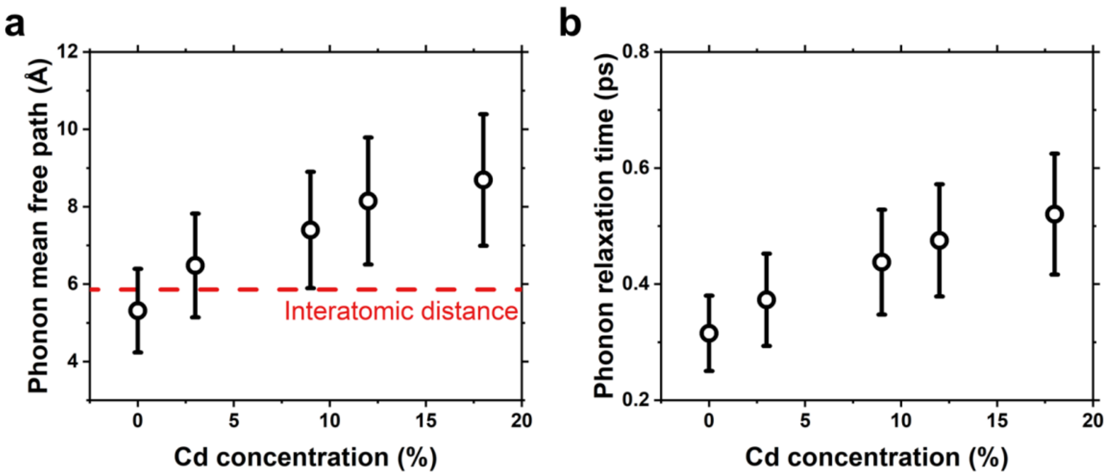

Supplementary Figure 8| (a) Estimated phonon mean free path of Cd-doped $AgSbSnSe_3$. The dashed red line denotes an interatomic distance of $AgSbSnSe_3$. (b) Calculated phonon relaxation time of Cd-doped $AgSbSnSe_3$.

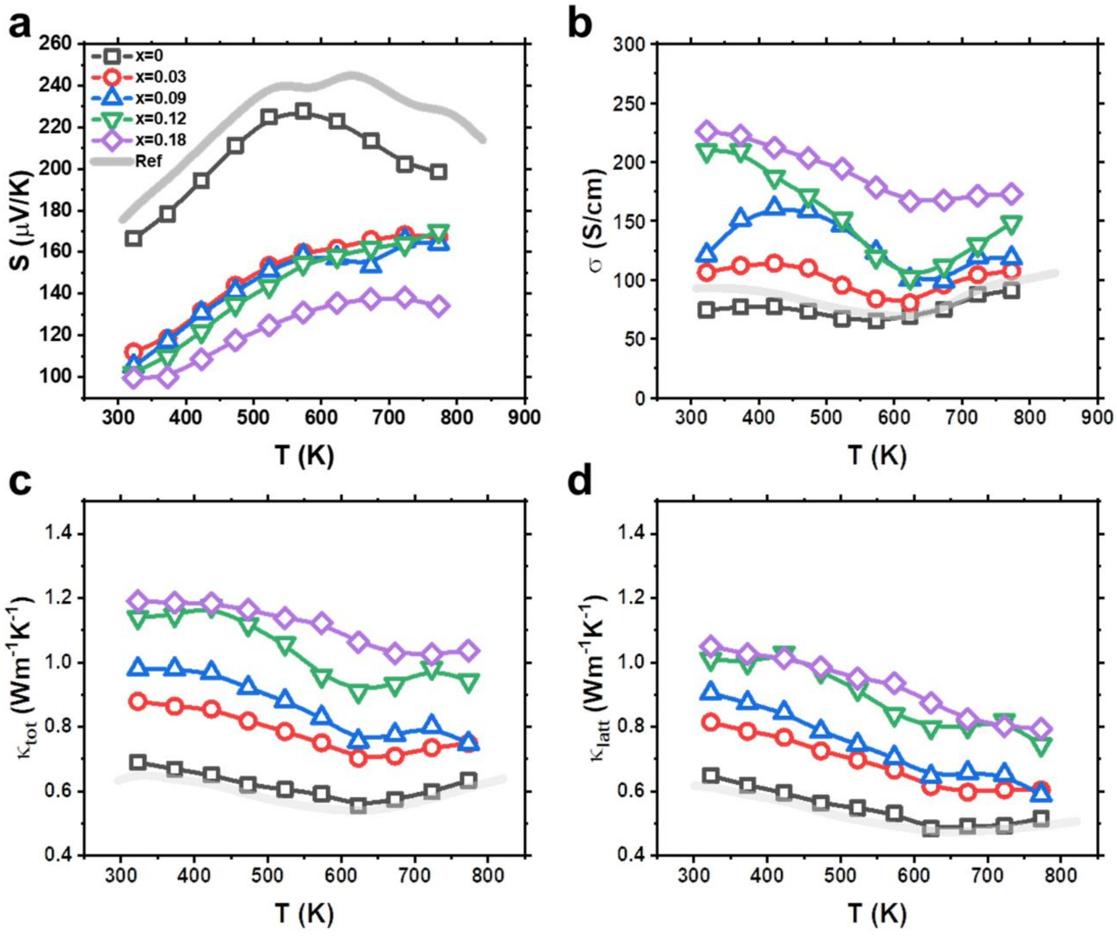

Supplementary Figure 9| Temperature-dependent thermoelectric properties. Temperature-dependent (a) Seebeck coefficient, (b) electrical conducticity, (c) total thermal conductivity, (d) lattice thermal conductivity of $AgSb_{1-x}Cd_xSnSe_3$. Measured properties of pristine $AgSbSnSe_3$ are consistent with those of the reference. The thermally activated charge transport observed in (b) near 450 K may originate from weak dielectric screening at the grain boundaries. The positive slope of electrical conductivity at 600 K is presumed to be the signature of cation disordering observed in ABX2-SnSe thermoelectrics.

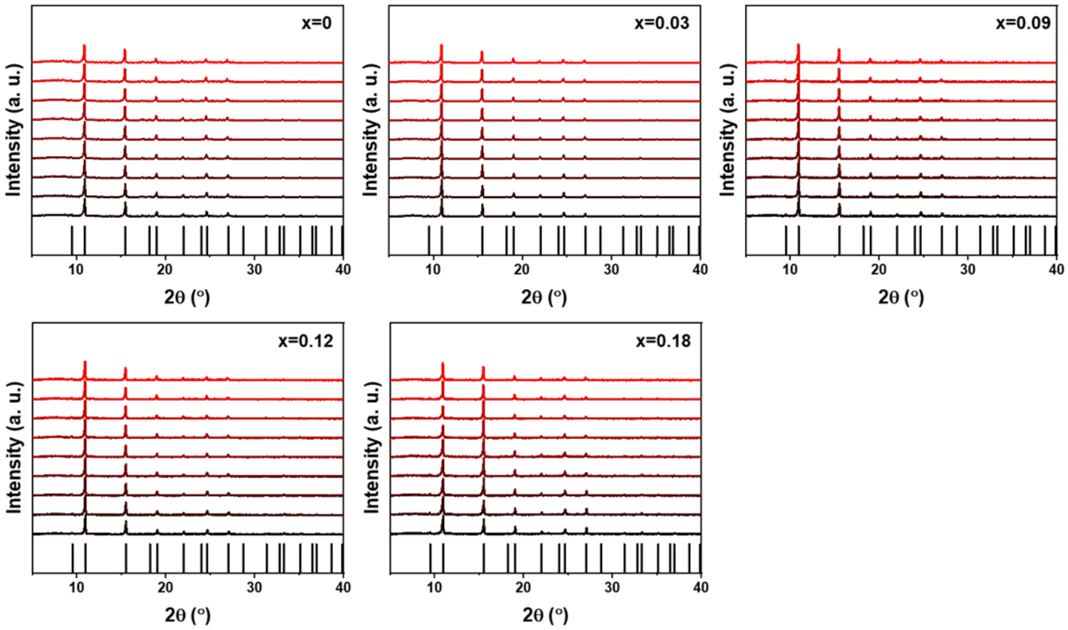

Supplementary Figure 10| Temperature-dependent XRD spectra for $AgSb_{1-x}Cd_xSnSe_3$. The temperature ranges from 298 K to 498 K with step interval of 25 K.

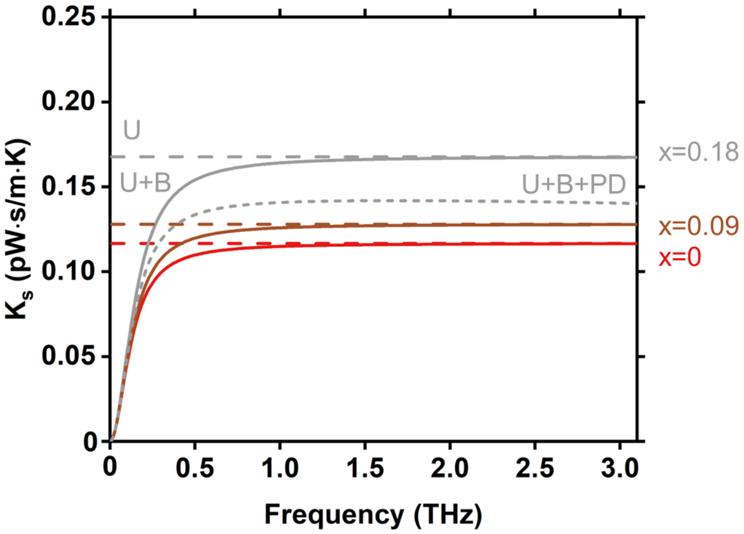

Supplementary Figure 11| Spectral thermal conductivity. Considering additional point defect scattering process significantly underestimate the lattice thermal conductivity, yet overall tendency is still dominated by Umklapp process.

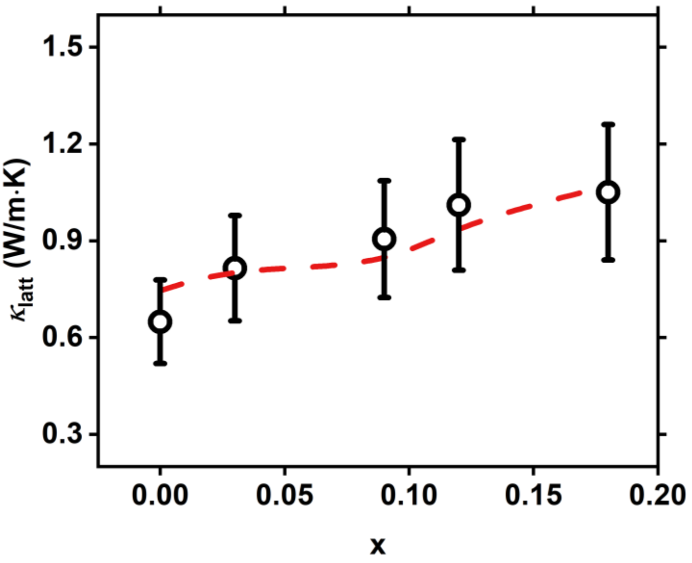

Supplementary Figure 12| Debye-Callaway model for estimating theoretical lattice thermal conductivity. Dashed red line denotes the theoretical lattice thermal conductivity estimated from modified Debye-Callaway model, while dots are experimentally measured values. Error bar denotes typical error (~20%) of lattice thermal conductivity estimation.

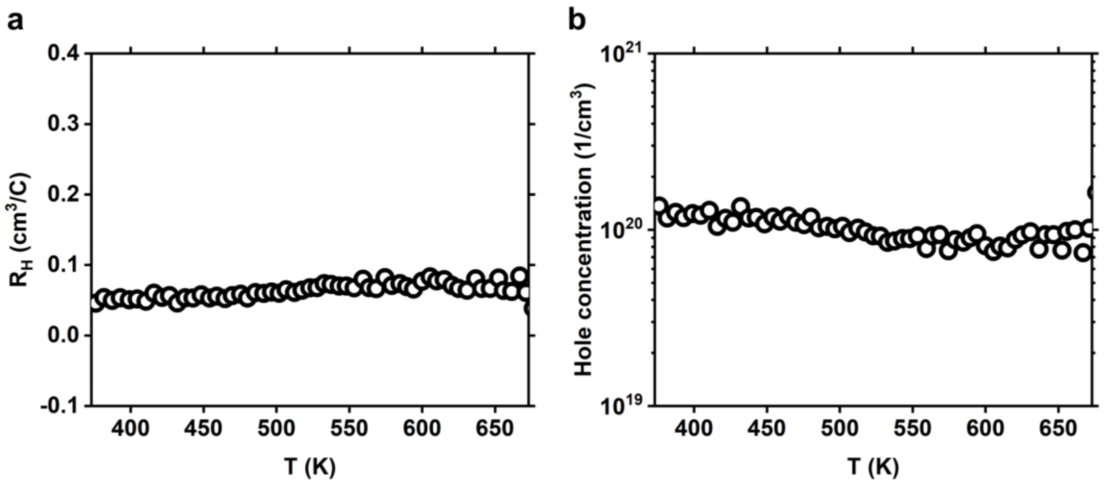

Supplementary Figure 13| High-temperature Hall measurement. (a) Hall coefficient and (b) corresponding hole concentration of $AgSnSb_{0.82}Cd_{0.18}Se_3$.

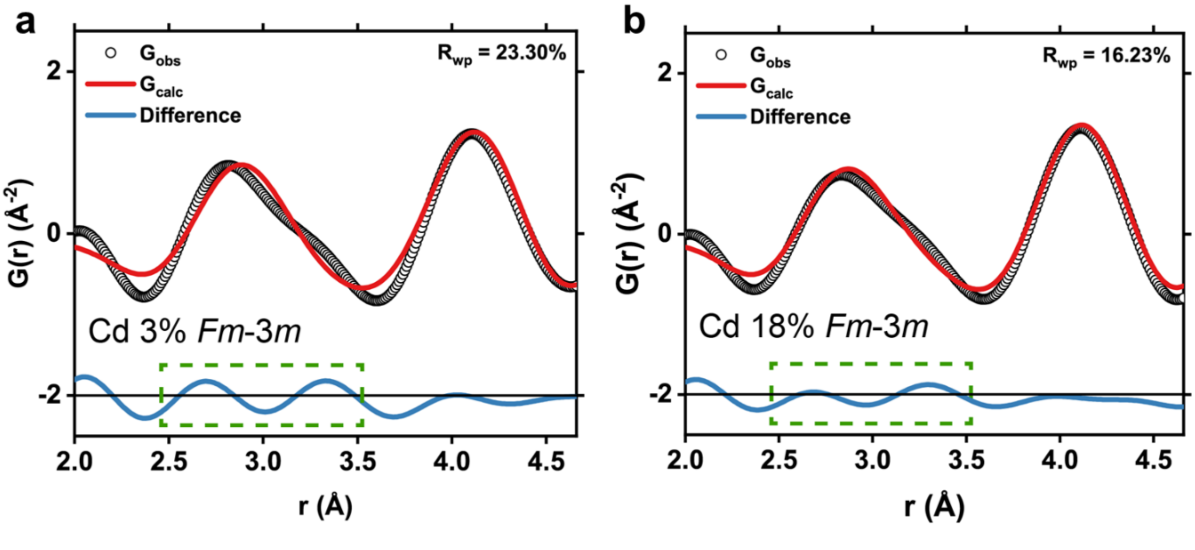

Supplementary Figure 14| Comparison of PDF fitting results of (a) $AgSnSb_{0.97}Cd_{0.03}Se_3$ and (b) $AgSnSb_{0.82}Cd_{0.18}Se_3$. Fitting was performed with the *Fm*-3*m* structure in both cases, where higher Cd concentration gives lower R_{wp} value because of less significant peak asymmetry.

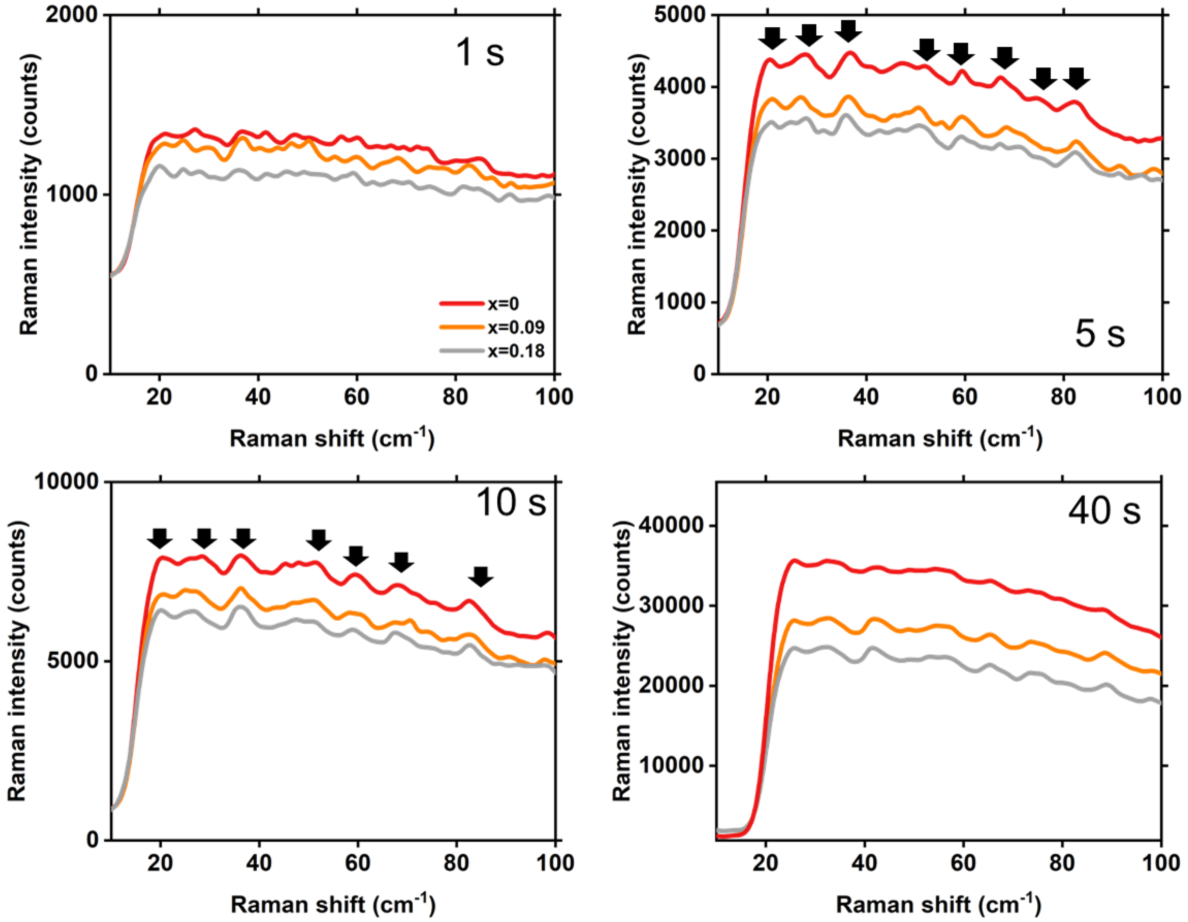

Supplementary Figure 15| Typical Raman spectra of $AgSnSb_{1-x}Cd_xSe_3$ (x=0, 0.09, 0.18) at room temperature.

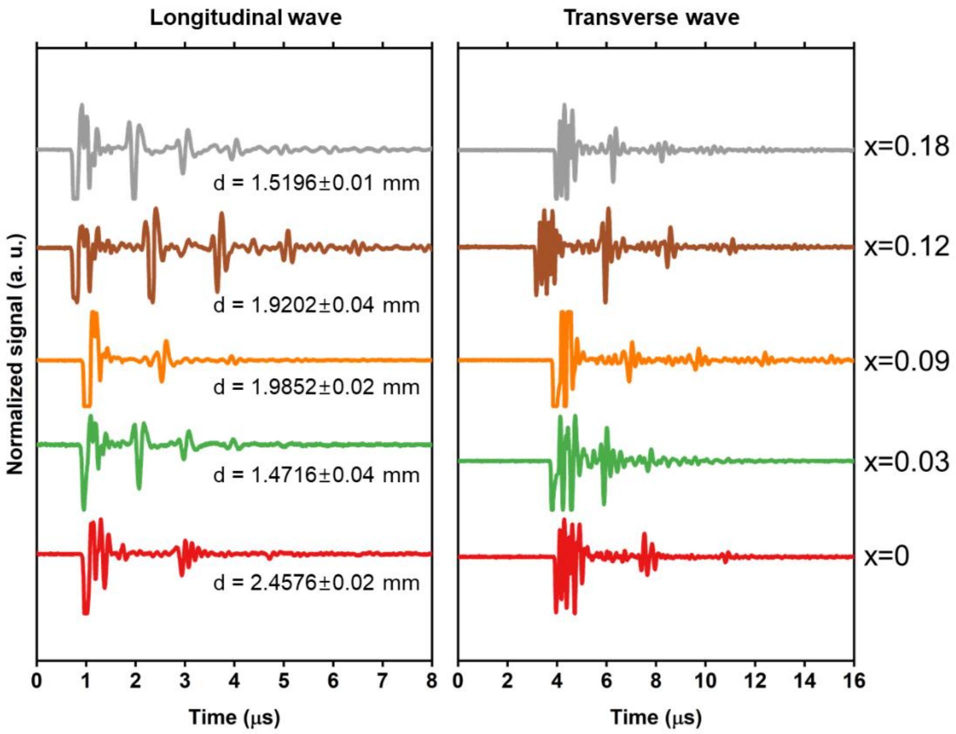

Supplementary Figure 16| Raw data of sound velocity measurement using ultrasonic pulse-echo method. The thickness of the sample (d) for longitudinal and transverse sound velocity measurement is same.

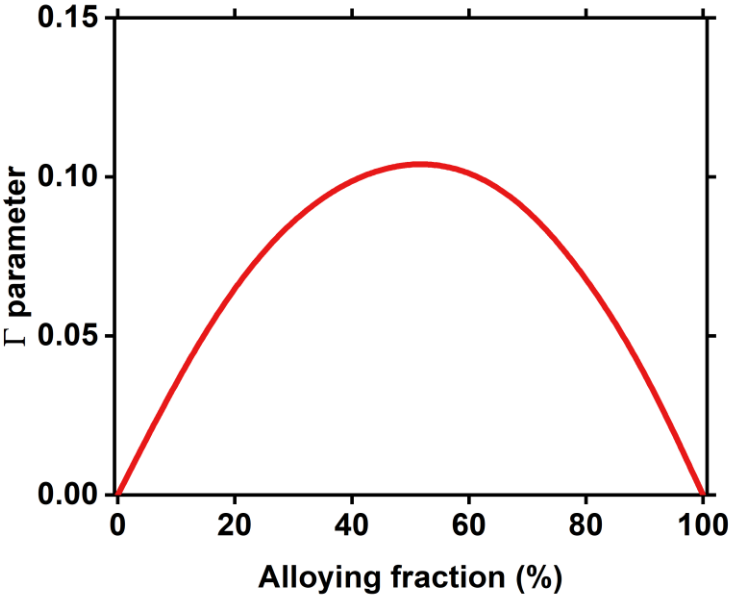

Supplementary Figure 17| Point defect scattering parameter used in Klemens alloy scattering model of $AgSbSnSe_{3-x}Te_x$.

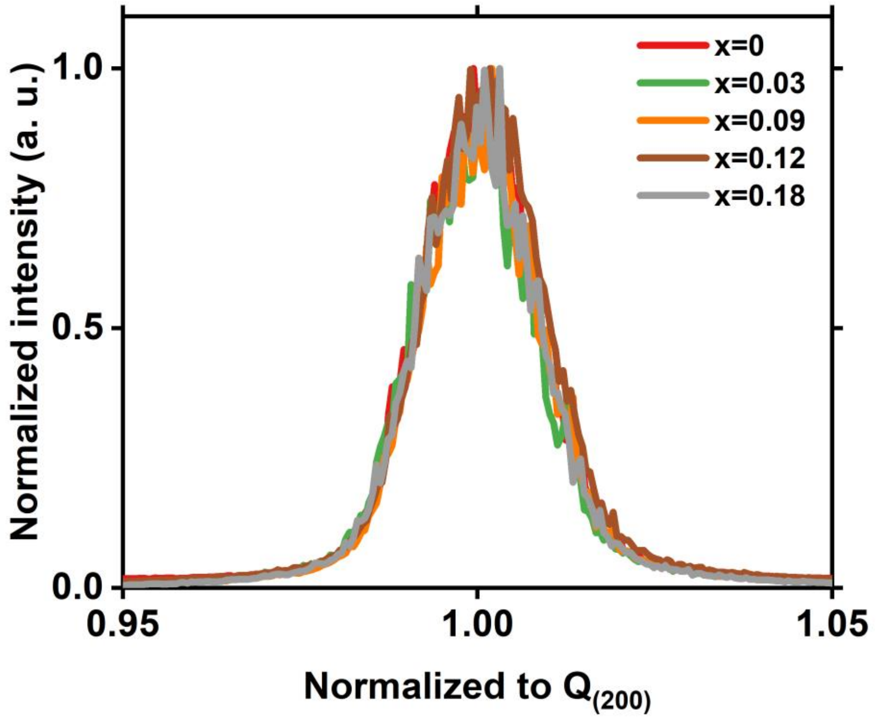

Supplementary Figure 18| Magnified image of normalized PXRD pattern of (200) diffraction.

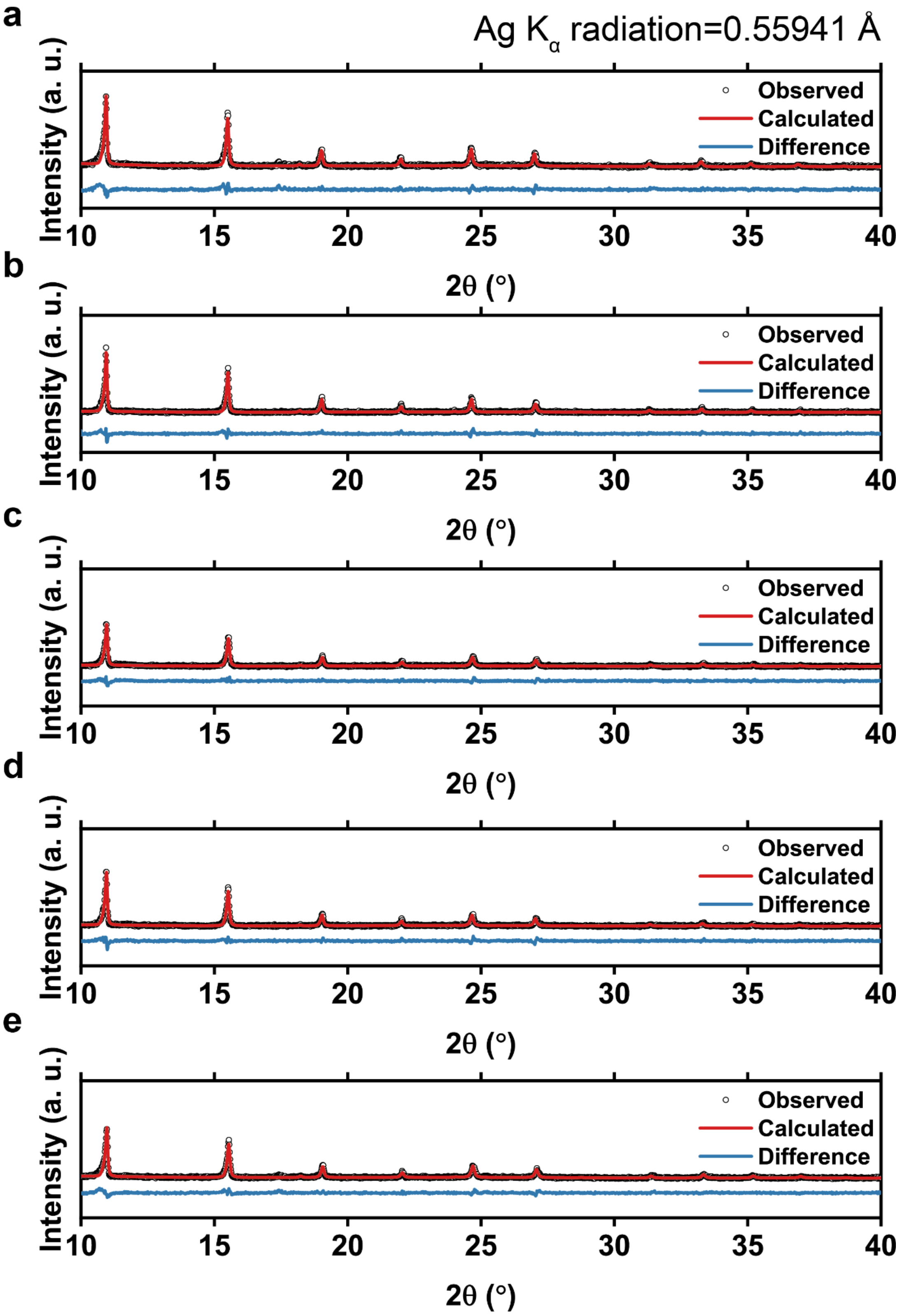

Supplementary Figure 19| Powder XRD pattern of $AgSnSb_{1-x}Cd_xSe_3$. PXRD pattern for (a) x=0, (b) x=0.03, (c) x=0.09, (d)x=0.12, (e) x=0.18 is shown with the observed and theoretical XRD pattern.

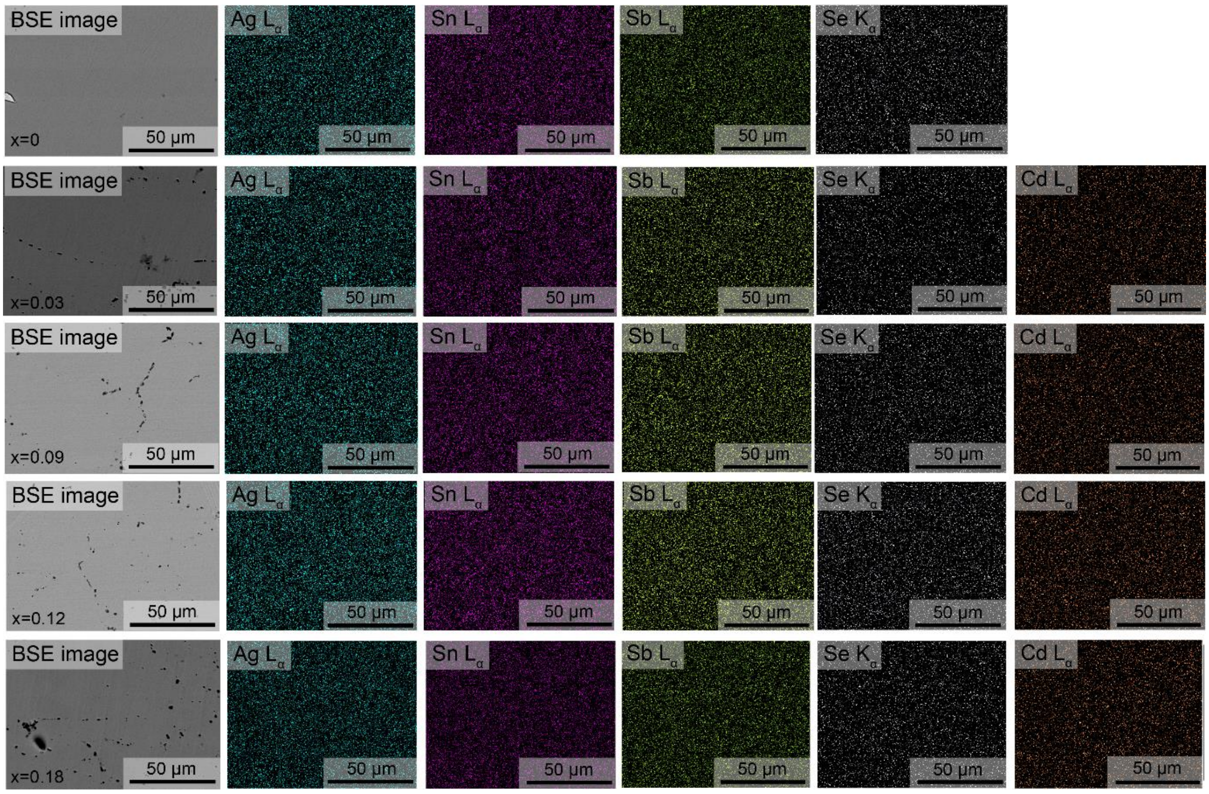

Supplementary Figure 20| Backscattered electron images of $AgSnSb_{1-x}Cd_xSe_3$ with corresponding EDS mapping. Homogeneous distribution of elements without formation of the secondary phase was observed.

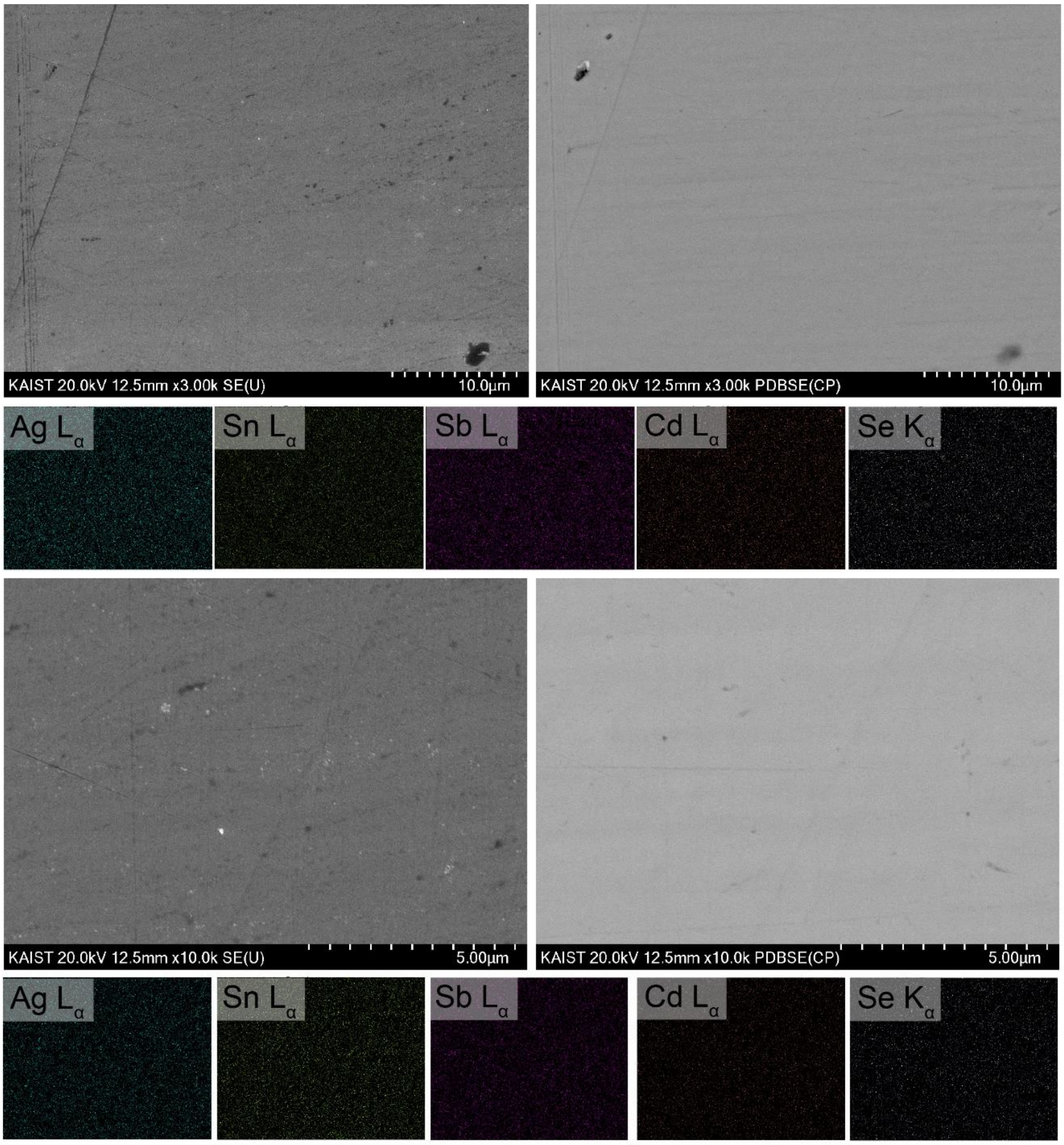

Supplementary Figure 21| SEM images of x=0.18 sample acquired using secondary electron (SE) detector and photodiode-backscattered electron (PDBSE) detector at different magnifications and corresponding EDS mapping results.

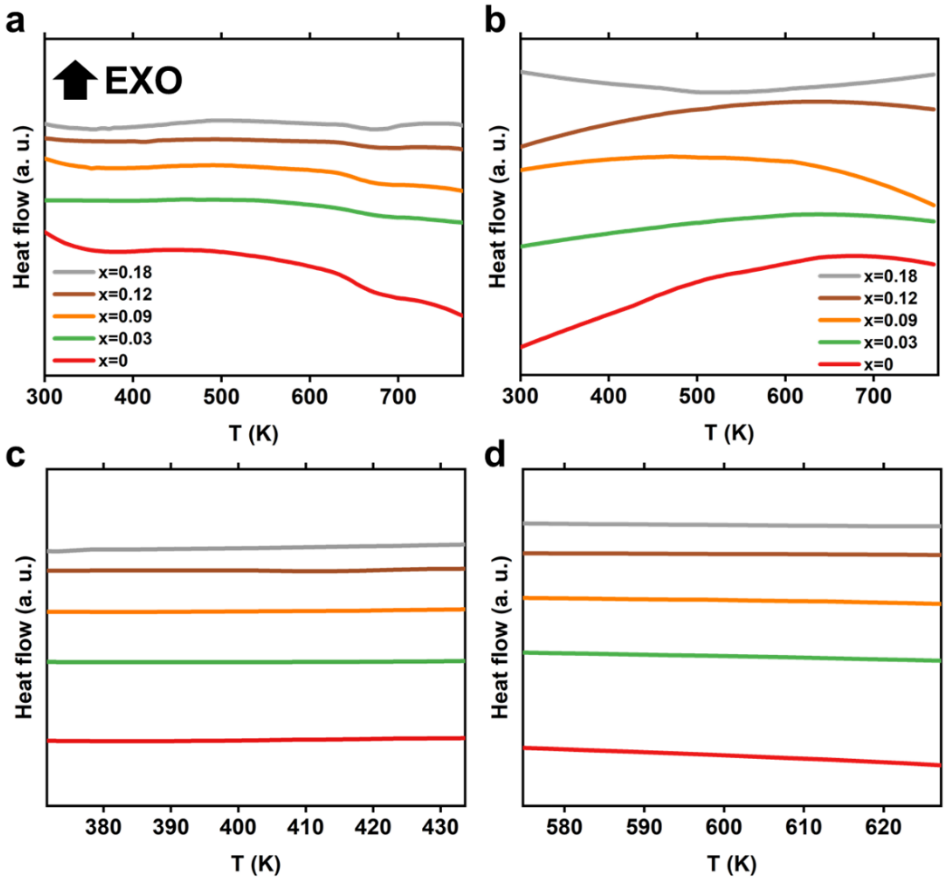

Supplementary Figure 22| Differential scanning calorimetry (DSC) results of $AgSnSb_{1-x}Cd_xSe_3$ for (a) heating and (b) cooling process under Ar gas. Magnified DSC profile near (c) 400 K and (d) 600 K are shown.

Supplementary Table 1| Fitted chemical potentials. Elemental chemical potentials (μ_i^0) that are fitted to experimental formation enthalpies of compounds in the Ag-Sb-Se-Cd phase space.

Element	μ_i^0 (eV)
Ag	-0.635

Sb	-3.654
Se	-3.847
Cd	-0.808

Supplementary Table 2| Extrinsic chemical potentials for different equilibrium phases. Extrinsic chemical potentials ($\Delta\mu_i$) of each element in all phase regions in the Ag-Sb-Se-Cd chemical space where $AgSbSe_2$ is stable.

Equilibrium phases	$\Delta\mu_{Ag}$ (eV)	$\Delta\mu_{Sb}$ (eV)	$\Delta\mu_{Se}$ (eV)	$\Delta\mu_{Cd}$ (eV)
Ag_2Se, CdSe, Se	-0.225	-0.7	0.0	-1.047
Sb_2Se_3, CdSe, Se	-0.258	-0.668	0.0	-1.047
Ag_3Sb, CdSe, Sb_2Se_3	-0.177	-0.423	-0.163	-0.884
Ag_3Sb, Ag_2Se, CdSe	-0.155	-0.488	-0.141	-0.906

Calculation of theoretical lattice thermal conductivity

The theoretical lattice thermal conductivity is calculated based on the modified Debye-Callaway model:

$$\kappa_{latt} = \frac{k_B}{2\pi^2 \upsilon}\left(\frac{k_B T}{\hbar}\right)^3 \int_0^{\frac{\theta_D}{T}} \tau(z)\frac{z^4 \exp(z)}{[\exp(z)-1]^2} dz,$$

,where υ is the average sound velocity and θ_D is Debye temperature of $AgSbSnSe_3$. For determination of relaxation time, Umklapp process, normal process, and grain boundary scattering is considered in the calculation. The relaxation time by Umklapp and normal process is given as follows:

$$\tau_U^{-1} + \tau_N^{-1} = \beta \frac{2}{(6\pi^2)^{1/3}} \frac{k_B \bar{V}^{1/3} \gamma^2 \omega^2 T}{\bar{M} \upsilon^3}$$

Sound velocities were experimentally determined from ultrasound measurements. Experimental Grüneisen parameter measured in this work was used for the calculation. Debye temperature was estimated from elastic properties by the equation $\theta_D = \frac{h}{k_B}\left[\frac{3q}{4\pi}\frac{N\rho}{M}\right]^{1/3} \upsilon$, where h is Planck constant, k_B is Boltzmann constant, q is the number of atoms in the unit cell, N is Avogadro's number, ρ is the bulk density, M is the molecular weight, and υ is the average sound velocity (J. Phys. Chem. Solids 24, pp. 909-917 (1963)). The relaxation time by grain boundary and point defect scattering is given as follows:

$$\tau_B^{-1} = \frac{\mathrm{d}}{\upsilon} \text{ (Grain boundary scattering)}$$

$$\tau_{PD}^{-1} = \frac{\bar{V}\omega^4}{4\pi\upsilon^3}\Gamma \text{ (Point defect scattering)}$$

The scattering parameter for point defect is calculated according to the previously reported method [Appl. Phys. Lett. 94, 153101 (2009)]. However, we noticed that including point defect scattering process significantly underestimate the theoretical lattice thermal conductivity of Cd-doped $AgSbSnSe_3$.

Supplementary Table 3| Parameters used to calculate the lattice thermal conductivity using modified Debye-Callaway model

Symbols	Description	Value	
v	Average sound velocity	x=0	1686 m/s
		x=0.03	1737 m/s
		x=0.09	1689m/s
		x=0.12	1714 m/s
		x=0.18	1669 m/s
θ_D	Debye temperature	149 K	
$\overline{V}$	Average molar volume	2.51×10^{-29} m^3	
$\overline{M}$	Average atomic mass	1.62×10^{-25} kg	
γ	Grüneisen parameter	x=0	3.21
		x=0.03	3.16
		x=0.09	3.05
		x=0.12	2.84
		x=0.18	2.65
β	Pre-factor for incorporating Umklapp and normal process	1.2 (fitted)	

Estimation of lattice thermal conductivity of alloys using Klemens model

The theoretical lattice thermal conductivity of alloys was estimated using Klemens analytical model, where the lattice thermal conductivity of an alloy (κ_{alloy}) is given by the following equation:

$$\frac{\kappa_{alloy}}{\kappa_0} = \frac{tan^{-1}u}{u}$$

,where κ_0 is the lattice thermal conductivity of the pure solid without defects and u is the disorder parameter. The disorder parameter is determined from the elastic properties of the pure material and point defect scattering parameter as follows:

$$u^2 = \frac{(6\pi^5V^2)^{1/3}}{2k_Bv_s}\kappa_0\Gamma$$

, where V is the average volume per atom, k_B is the Boltzmann constant, v_s is the average sound velocity (1686 m/s), and Γ is the point defect scattering parameter. Γ is estimated from the two principal components: mass fluctuation (Γ_M) and strain field fluctuation (Γ_S), which is given as follows:

$$\Gamma_M + \Gamma_S = \frac{\langle\overline{\Delta M^2}\rangle}{\langle\overline{M}\rangle^2} + \varepsilon\frac{\langle\overline{\Delta R^2}\rangle}{\langle\overline{R}\rangle^2}$$

, where $\langle\overline{M}\rangle$ is the average atomic mass, $\langle\overline{\Delta M}\rangle$ is the average atomic mass variation, $\langle\overline{R}\rangle$ is the average atomic radius of the site, $\langle\overline{\Delta R}\rangle$ is the average atomic radius variation, and ε is the phenomenological parameter for the strain fluctuation that typically varies from 1 to 500 to fit the experimental data.

Notes on the effect of intrinsic point defects (V_{Ag} and Ag_{Sb})

It can be seen that some intrinsic defects (e.g., V_{Ag} or Ag_{Sb}) are observed that have lower formation energy than that of Cd_{Sb}. However, given that these intrinsic point defects are present regardless of the Cd content, their effect on the lattice thermal conductivity should be present regardless of Cd doping, while the effect of V_{Ag} and Ag_{Sb} is not immediately clear.

Furthermore, suppose V_{Ag} formation becomes more pronounced with Cd doping. As we already discussed in the main text, the formation of V_{Ag} would not affect the LPE concentration considering the electron configuration of Ag. However, the increased point defect scattering due to mass fluctuation (zero mass *versus* matrix) would reduce the lattice thermal conductivity, which cannot explain the experimentally observed rise of the lattice thermal conductivity. We might exclude the possibility of the formation of Ag_{Sb} as well to see the decrease in the lattice parameter. The ionic radius of Ag^+ and Sb^{3+} is 115 pm and 76 pm, respectively. If the antisite defect is dominating in $AgSnSbSe_3$, we should observe an increase in the lattice parameter. Therefore, neither V_{Ag} nor Ag_{Sb} are significantly affecting the properties of Cd-doped $AgSbSnSe_3$.

Supplementary Table 4| Structural data of $AgSnSb_{1-x}Cd_xSe_3$ obtained from the Rietveld refinement of PXRD pattern

$AgSnSbSe_3$<br>Space group: *Fm*-3*m*<br>a = 5.85873 Å<br>R_w = 13.291%					
Atom	x	y	z	Occupancy	U_{iso}/Å^2
Ag	0.5	0.5	0.5	0.3333	0.04461
Sb	0.5	0.5	0.5	0.3333	0.04461
Sn	0.5	0.5	0.5	0.3333	0.04461

Se	0	0	0	1.0000	0.02679

$AgSnSb_{0.97}Cd_{0.03}Se_3$<br><br>Space group: *Fm*-3*m*<br><br>a = 5.85482 Å<br><br>R_w = 16.264 %					
Atom	x	y	z	Occupancy	U_{iso}/Å^2
Ag	0.5	0.5	0.5	0.3333	0.04105
Sb	0.5	0.5	0.5	0.3233	0.04105
Cd	0.5	0.5	0.5	0.0100	0.04105
Sn	0.5	0.5	0.5	0.3333	0.04105
Se	0	0	0	1.0000	0.02611

$AgSnSb_{0.91}Cd_{0.09}Se_3$<br><br>Space group: *Fm*-3*m*<br><br>a = 5.850299 Å<br><br>R_w = 16.636 %

Atom	x	y	z	Occupancy	U_{iso}/Å^2
Ag	0.5	0.5	0.5	0.3333	0.03769
Sb	0.5	0.5	0.5	0.3033	0.03769
Cd	0.5	0.5	0.5	0.0300	0.03769
Sn	0.5	0.5	0.5	0.3333	0.03769
Se	0	0	0	1.0000	0.02799

$AgSnSb_{0.88}Cd_{0.12}Se_3$<br><br>Space group: *Fm*-3*m*<br><br>a = 5.84785 Å<br><br>R_w = 16.270 %					
Atom	x	y	z	Occupancy	U_{iso}/Å^2
Ag	0.5	0.5	0.5	0.3333	0.03610
Sb	0.5	0.5	0.5	0.2933	0.03610
Cd	0.5	0.5	0.5	0.0400	0.03610
Sn	0.5	0.5	0.5	0.3333	0.03610
Se	0	0	0	1.0000	0.02705

$AgSnSb_{0.82}Cd_{0.18}Se_3$<br>Space group: *Fm*-3*m*<br>a = 5.84232 Å<br>R_w = 15.843 %					
Atom	x	y	z	Occupancy	U_{iso}/Å^2
Ag	0.5	0.5	0.5	0.3333	0.03582
Sb	0.5	0.5	0.5	0.2733	0.03582
Cd	0.5	0.5	0.5	0.0600	0.03582
Sn	0.5	0.5	0.5	0.3333	0.03582
Se	0	0	0	1.0000	0.02694

Supplementary Table 5| Actual and relative density of $AgSnSb_{1-x}Cd_xSe_3$ samples

Composition	Density (g/cm^3)	Relative density (%)
$AgSnSbSe_3$	6.27720	97.5
$AgSnSb_{0.97}Cd_{0.03}Se_3$	6.35206	98.6
$AgSnSb_{0.91}Cd_{0.09}Se_3$	6.30405	98.0

$AgSnSb_{0.88}Cd_{0.12}Se_3$	6.18831	96.2
$AgSnSb_{0.82}Cd_{0.18}Se_3$	6.23054	96.4
